# Supplementary material for: Nanostructured Polyelectrolyte Complexes Based on Water-Soluble Thiacalix[4]Arene and Pillar[5]Arene: Self-Assembly in Micelleplexes and Polyplexes at Packaging DNA
Source: Nanomaterials (Basel). 2020 Apr 17;10(4):777. doi: 10.3390/nano10040777 (PMC7221682; doi:10.3390/nano10040777)
Supplement: Supplementary file 1 [file nanomaterials-10-00777-s001.pdf]

nanomaterials

Supplementary Materials

Nanostructured Polyelectrolyte Complexes Based on Water-Soluble Thiacalix[4]Arene and Pillar[5]Arene: Self-Assembly in Micelleplexes and Polyplexes at Packaging DNA

Luidmila S. Yakimova *, Aigul R. Nugmanova, Olga A. Mostovaya, Alena A. Vavilova, Dmitriy N. Shurpik, Timur A. Mukhametzyanov and Ivan I. Stoikov *

Kazan Federal University, A.M. Butlerov Chemical Institute, 420008, Kremlevskaya Street, 18, Kazan and Russian Federation; aygul9pul9@mail.ru (A.R.N.); olga.mostovaya@mail.ru (O.A.M.); anelia_86@mail.ru (A.A.V.); DNShurpik@mail.ru (D.N.S.); timmie.m@gmail.com (T.A.M.)

* Correspondence: mila.yakimova@mail.ru (L.S.Y.); ivan.stoikov@mail.ru (I.I.S.); Tel.: +7-843 233-7241 (L.S.Y. and I.I.S.)

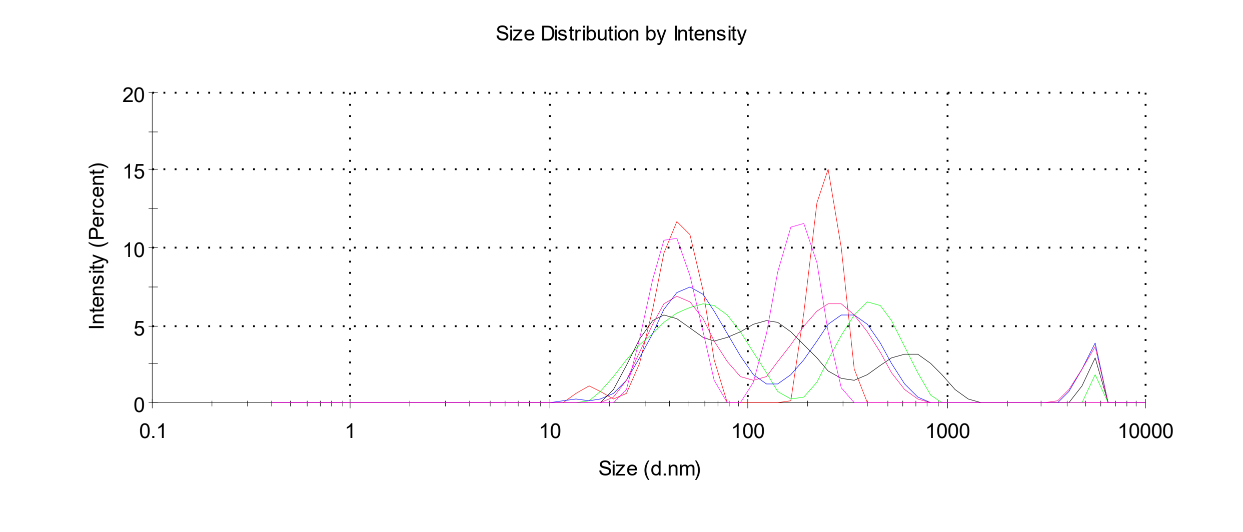

Figure S1. Size distribution of the co-interpolyelectrolyte associates AP[5]A/STC[4]A (1:1 molar ratio, the concentration of initial solutions AP[5]A and STC[4]A were 3 × 10^{-4} M, final volume was 1 mL). Each line in figure is one measurement from six.

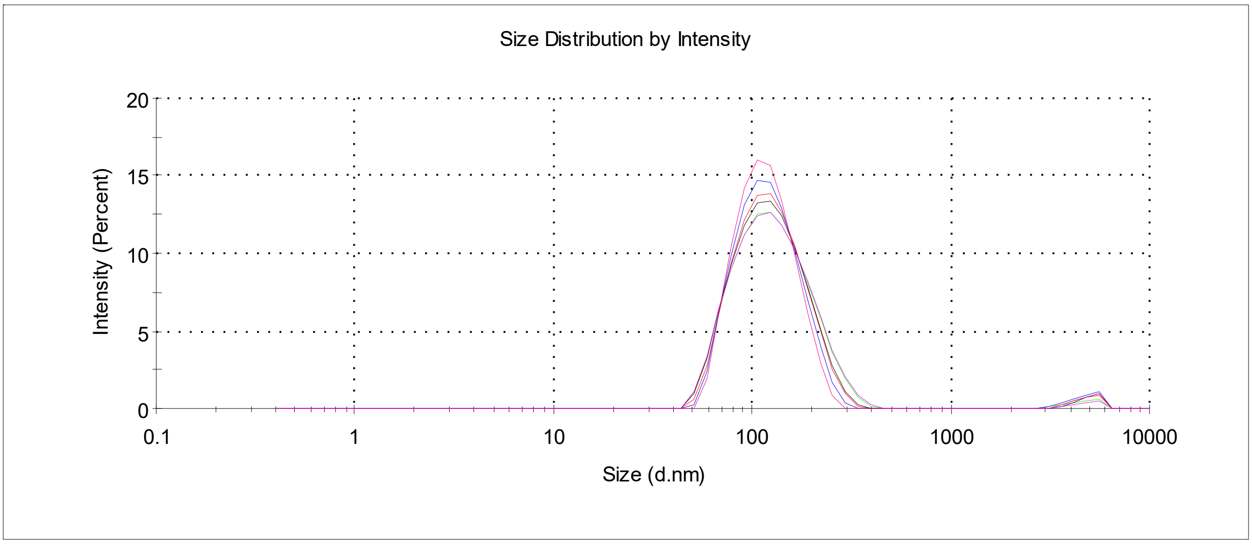

Figure S2. Size distribution of the co-interpolyelectrolyte associates AP[5]A/STC[4]A (1:2 molar ratio, the concentration of initial solutions AP[5]A and STC[4]A was 3 × 10^{-4} M, final volume was 1 mL). Each line in figure is one measurement from six.

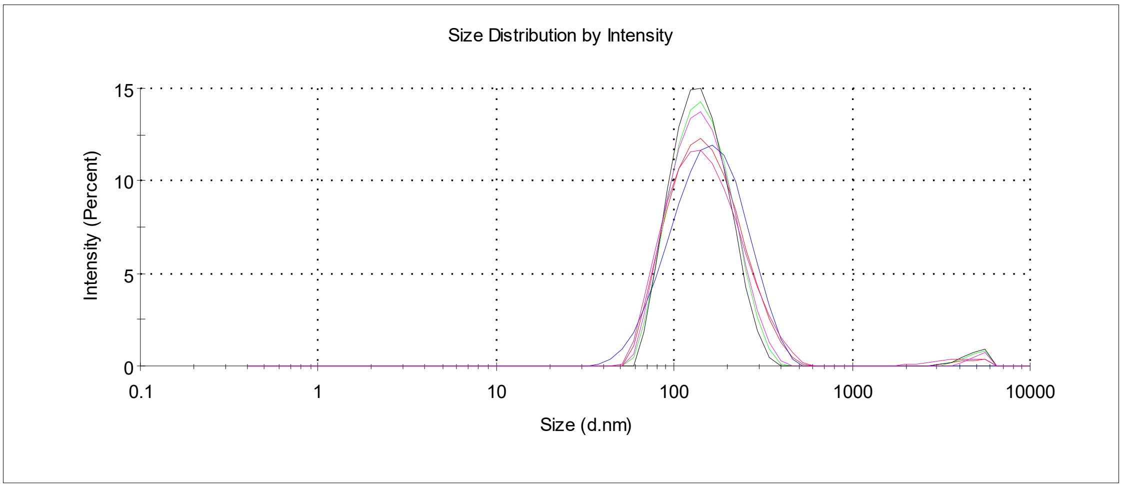

Figure S3. Size distribution of the co-interpolyelectrolyte associates AP[5]A/STC[4]A (1:2.5 molar ratio, the concentration of initial solutions AP[5]A and STC[4]A was 3 × 10^{-4} M, final volume was 1 mL). Each line in figure is one measurement from six.

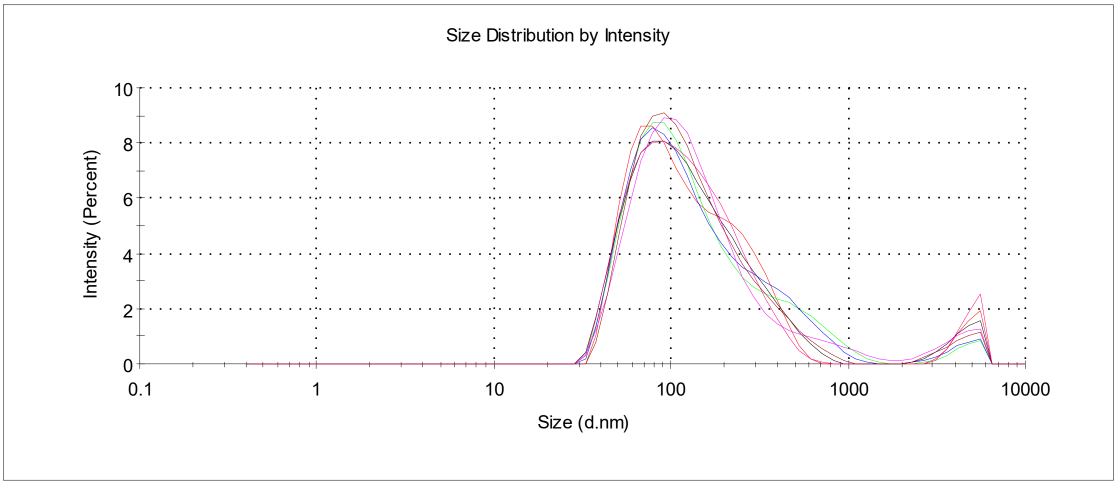

Figure S4. Size distribution of the co-interpolyelectrolyte associates AP[5]A/STC[4]A (1:3 molar ratio, the concentration of initial solutions AP[5]A and STC[4]A was 3 × 10^{-4} M, final volume was 1 mL). Each line in figure is one measurement from six.

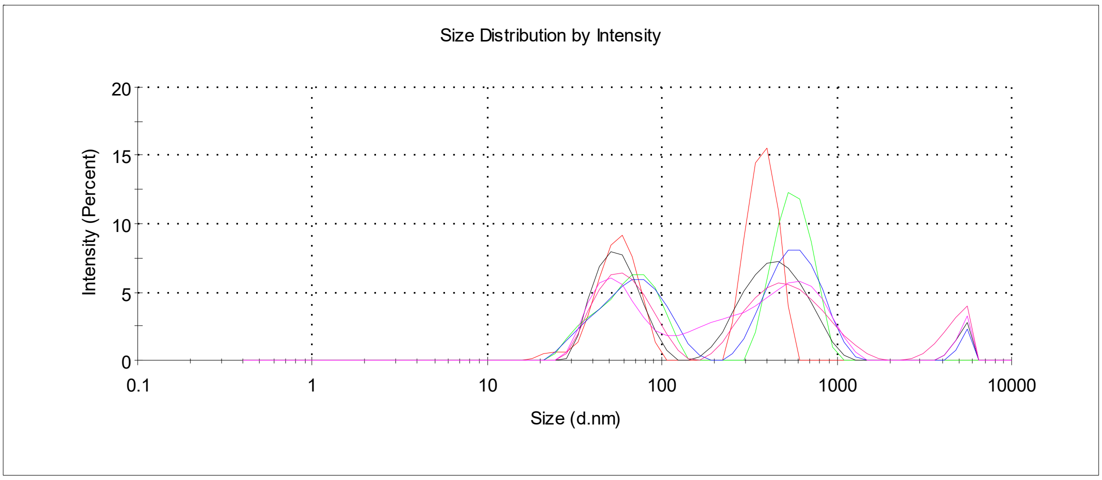

Figure S5. Size distribution of the co-interpolyelectrolyte associates AP[5]A/STC[4]A (1:9 molar ratio, the concentration of initial solutions AP[5]A and STC[4]A was 3 × 10^{-4} M, final volume was 1 mL). Each line in figure is one measurement from six.

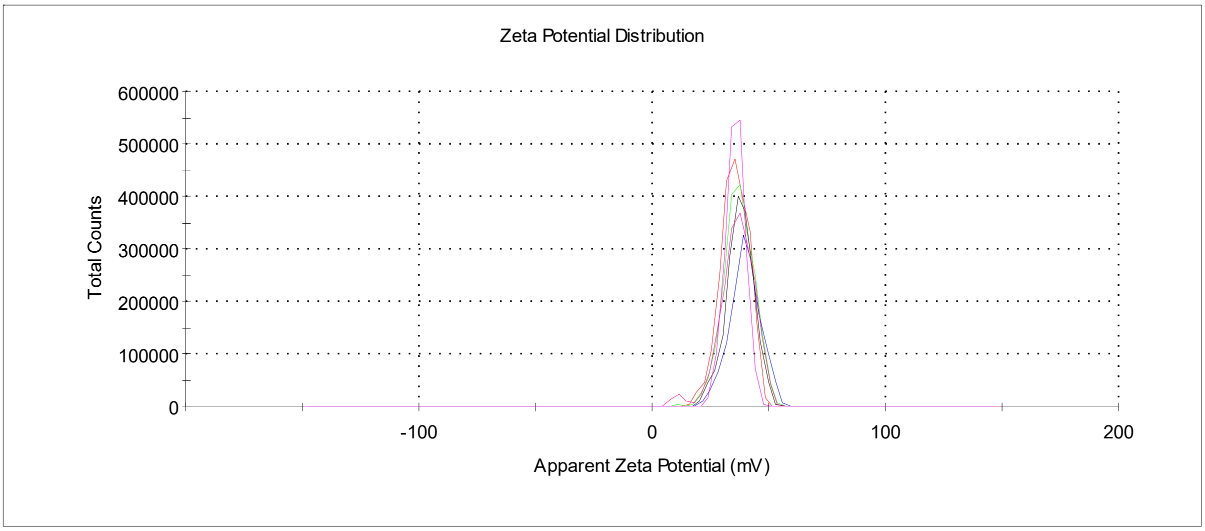

Figure S6. Zeta potential distributions of the associates between co-interpolyelectrolyte associate AP[5]A/STC[4]A (1:2 molar ratio, the concentration of initial solutions AP[5]A and STC[4]A are 3 × 10^{-4} M, final volume final volume was 1 mL). Each line in figure is one measurement from six.

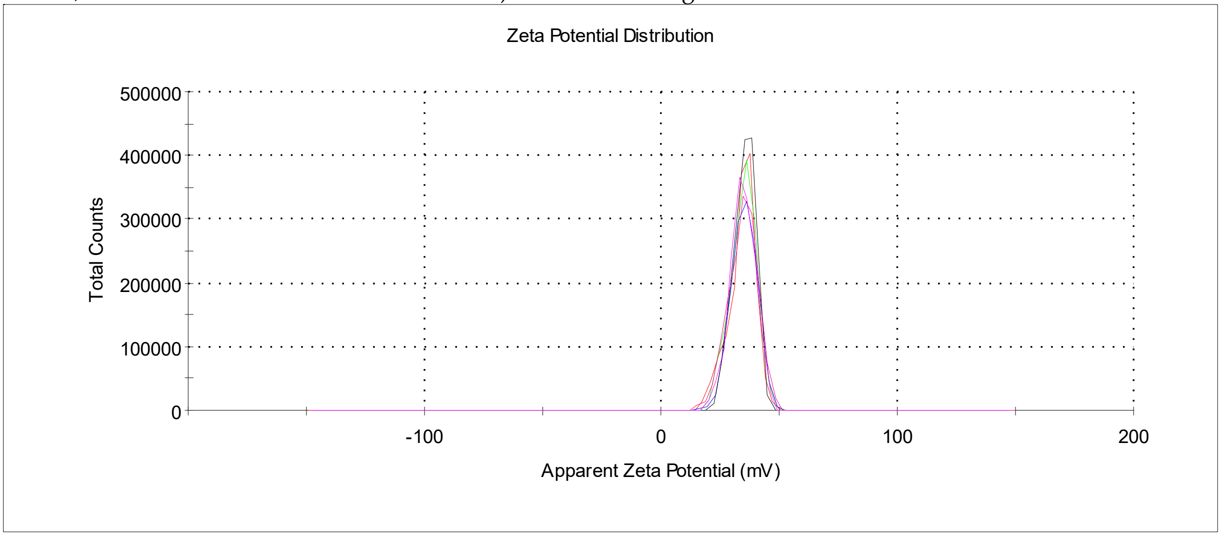

Figure S7. Zeta potential distributions of the associates between co-interpolyelectrolyte associate AP[5]A/STC[4]A (1:2.5 molar ratio, the concentration of initial solutions AP[5]A and STC[4]A was 3 × 10^{-4} M, final volume final volume was 1 mL). Each line in figure is one measurement from six.

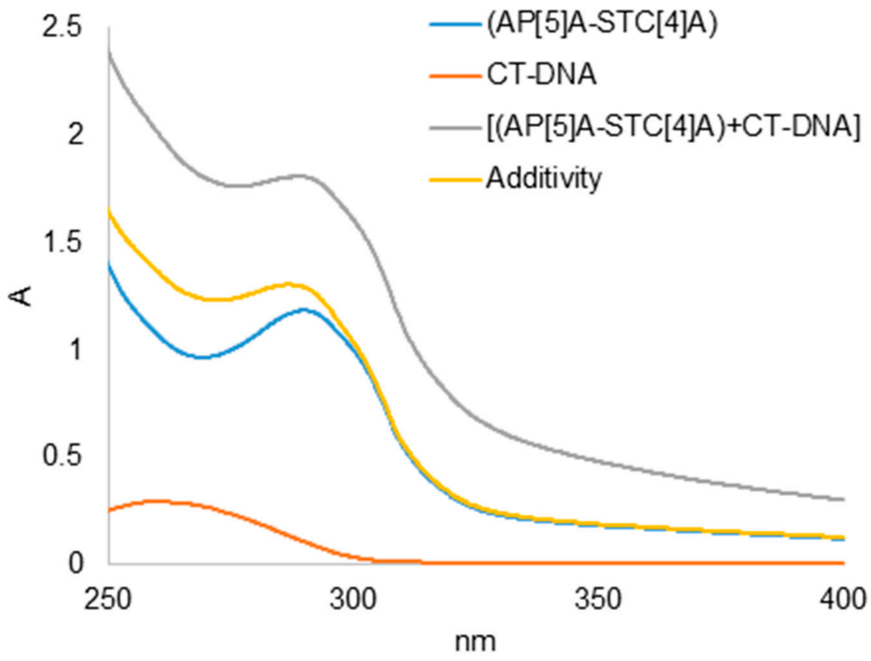

Figure S8. UV-vis spectra for co-interpolyelectrolyte associates AP[5]A/STC[4]A (1:2 molar ratio), CT-DNA, and mixture of co-interpolyelectrolyte associates AP[5]A/STC[4]A and CT-DNA in buffer at 1:3 molar ratio. Additivity is sum of two absorption spectra (AP[5]A/STC[4]A and CT-DNA). The concentration of CT-DNA was 1.6×10^{-4} M.

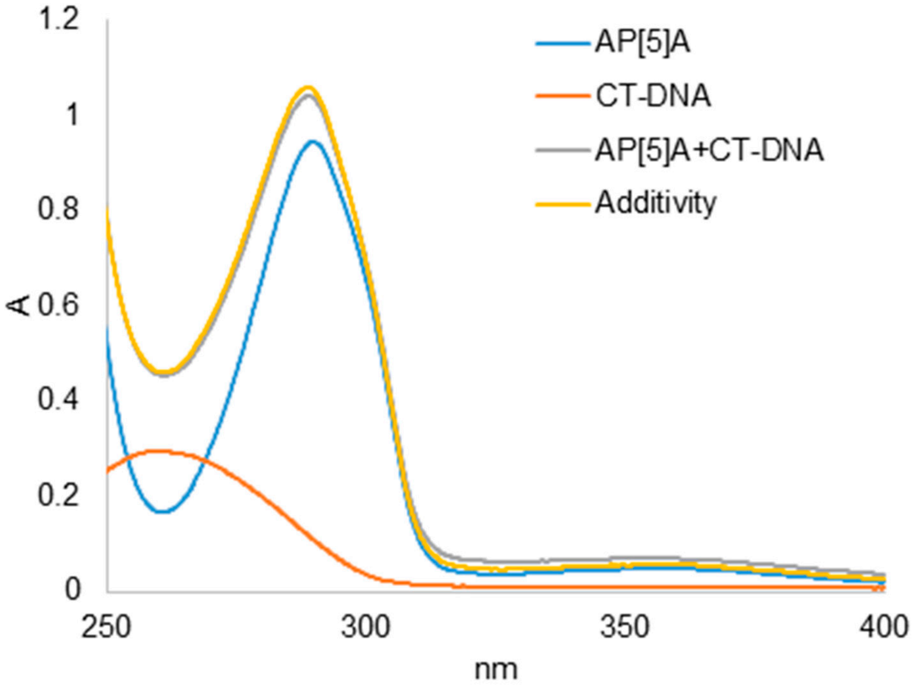

Figure S9. UV-VIS spectra for associates AP[5]A, CT-DNA, and mixture of AP[5]A and CT-DNA in buffer at 1:3 molar ratio. Additivity is sum of two absorption spectra (AP[5]A and CT-DNA). The concentration of CT-DNA was 1.6×10^{-4} M.

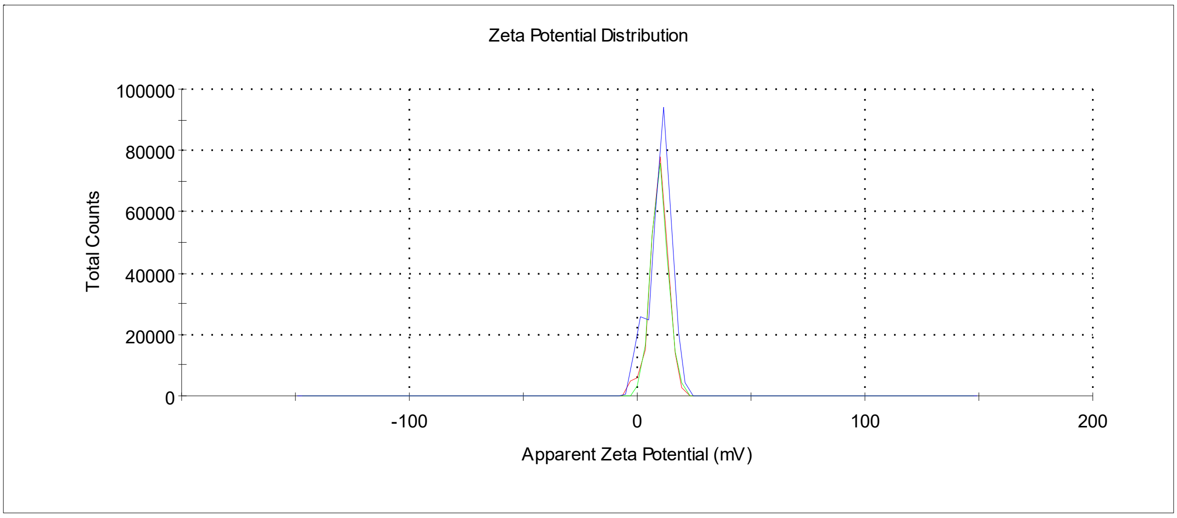

Figure S10. Zeta potential distributions of the associates between co-interpolyelectrolyte associate AP[5]A/STC[4]A (1:2.5 molar ratio, the concentration of initial solutions AP[5]A and STC[4]A was 3 × 10^{-6} M, final volume was 1 mL). Each line in Figure is one measurement from three.

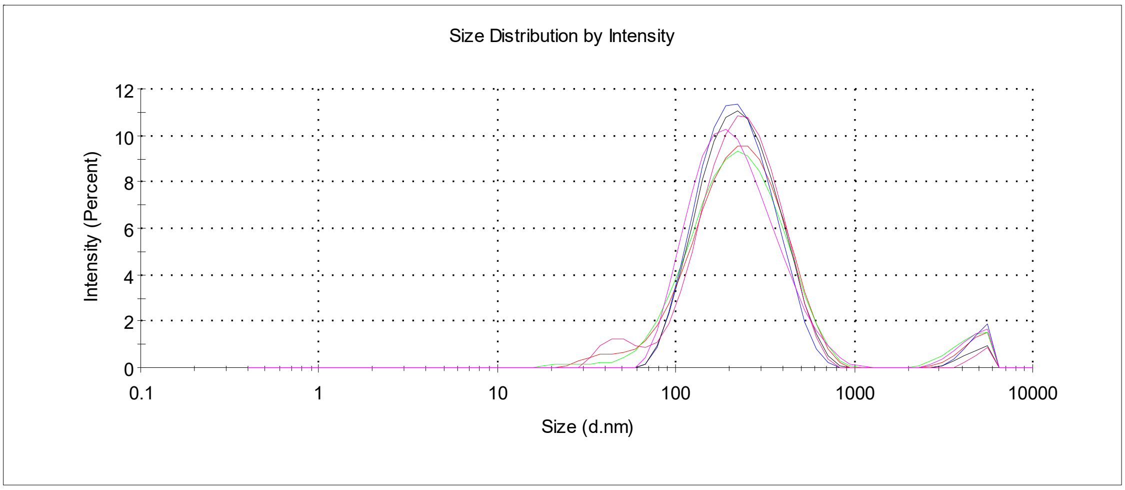

Figure S11. Size distribution of the associates between co-interpolyelectrolyte associate AP[5]A/STC[4]A (1:2 molar ratio) and CT-DNA at 1:10 molar ratio. The concentration of CT-DNA was 0.9 × 10^{-4} M. Each line in figure is one measurement from six.

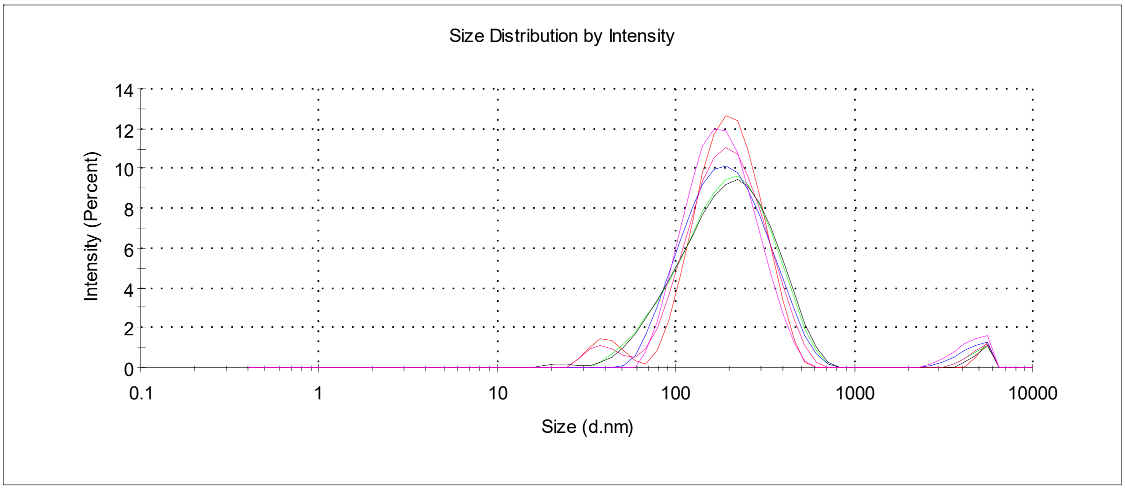

Figure S12. Size distribution of the associates between co-interpolyelectrolyte associate AP[5]A/STC[4]A (1:2 molar ratio) and CT-DNA at 1:7 molar ratio. The concentration of CT-DNA was 0.9 × 10^{-4} M. Each line in figure is one measurement from six.

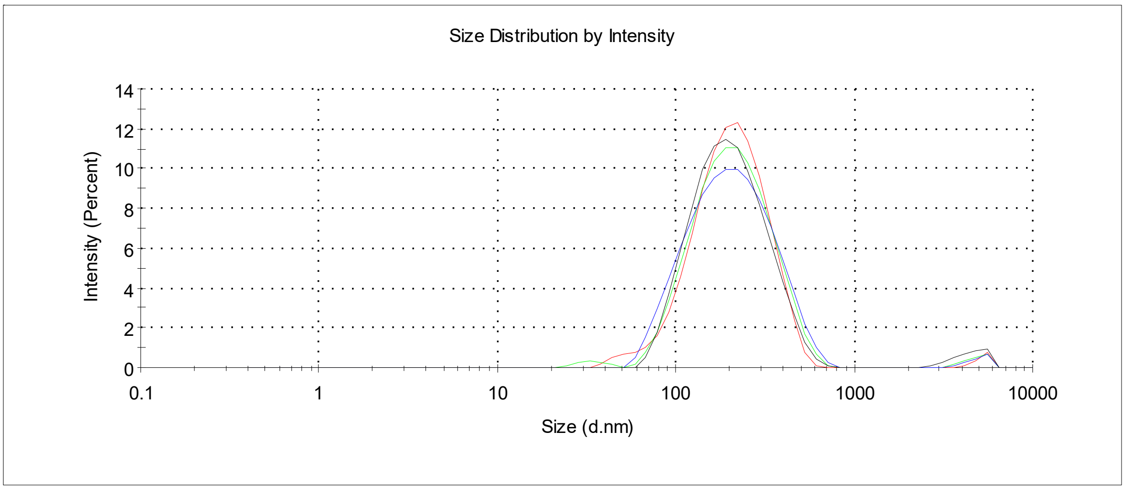

Figure S13. Size distribution of the associates between co-interpolyelectrolyte associate AP[5]A/STC[4]A (1:2 molar ratio) and CT-DNA at 1:3 molar ratio. The concentration of CT-DNA was 0.9 × 10^{-4} M. Each line in figure is one measurement from four.

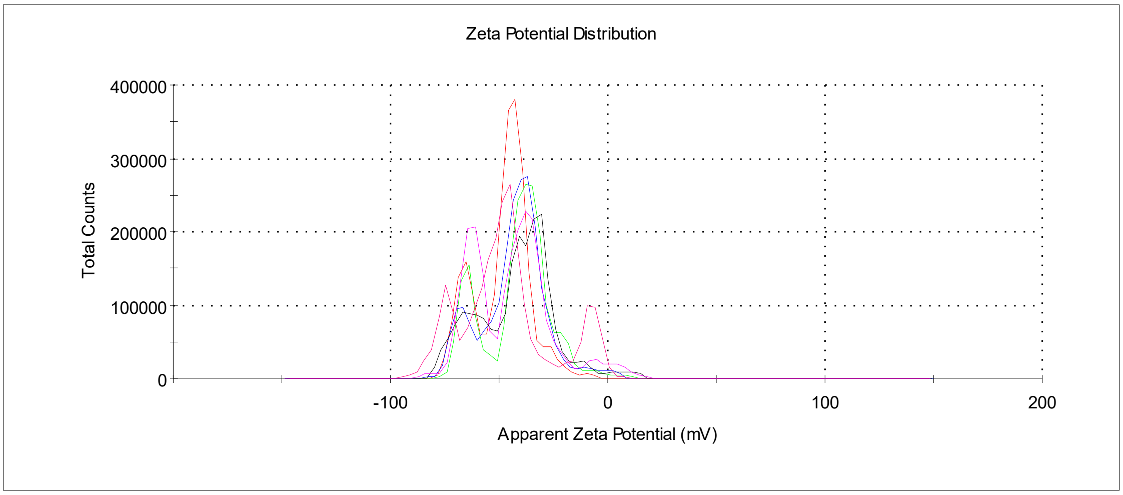

Figure S14. Zeta potential distributions of the associates between co-interpolyelectrolyte associate AP[5]A/STC[4]A (1:2 molar ratio) and CT-DNA at 1:10 molar ratio. The concentration of CT-DNA was 0.9 × 10^{-4} M. Each line in figure is one measurement from six.

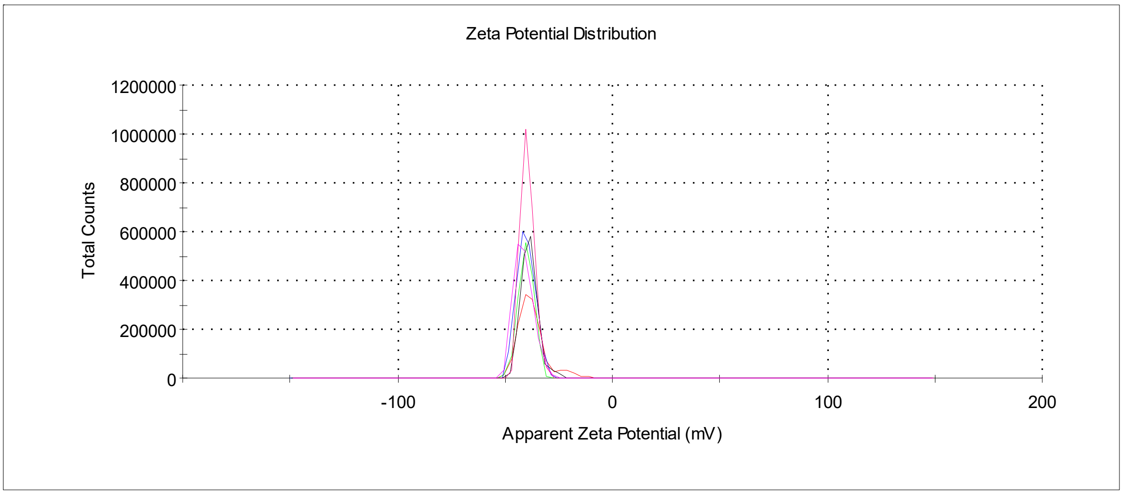

Figure S15. Zeta potential distributions of the associates between co-interpolyelectrolyte associate AP[5]A/STC[4]A (1:2 molar ratio) and CT-DNA at 1:7 molar ratio. The concentration of CT-DNA was 0.9 × 10^{-4} M. Each line in figure is one measurement from six.

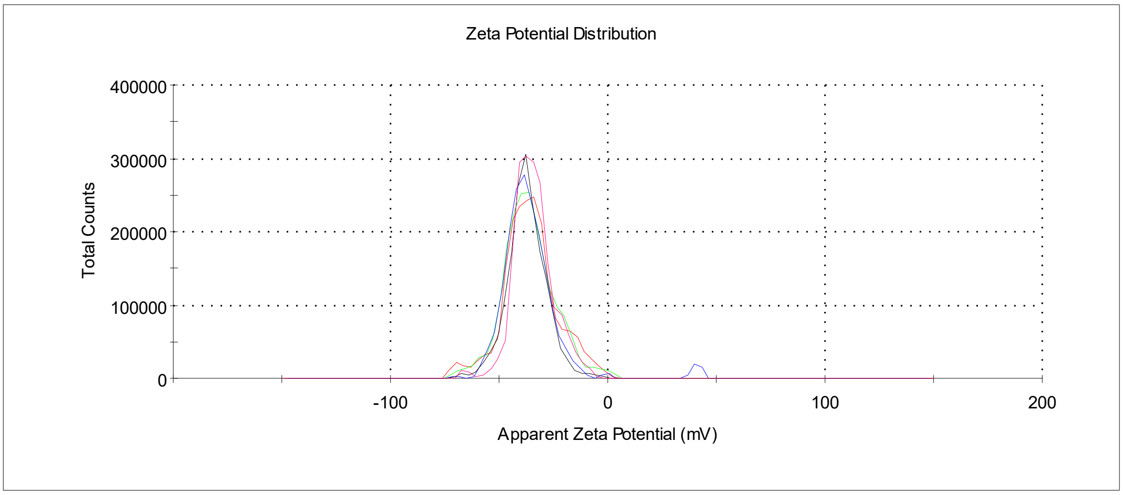

Figure S16. Zeta potential distributions of the associates between co-interpolyelectrolyte associate AP[5]A/STC[4]A (1:2 molar ratio) and CT-DNA at 1:3 molar ratio. The concentration of CT-DNA was 0.9×10^{-4} M. Each line in figure is one measurement from five.

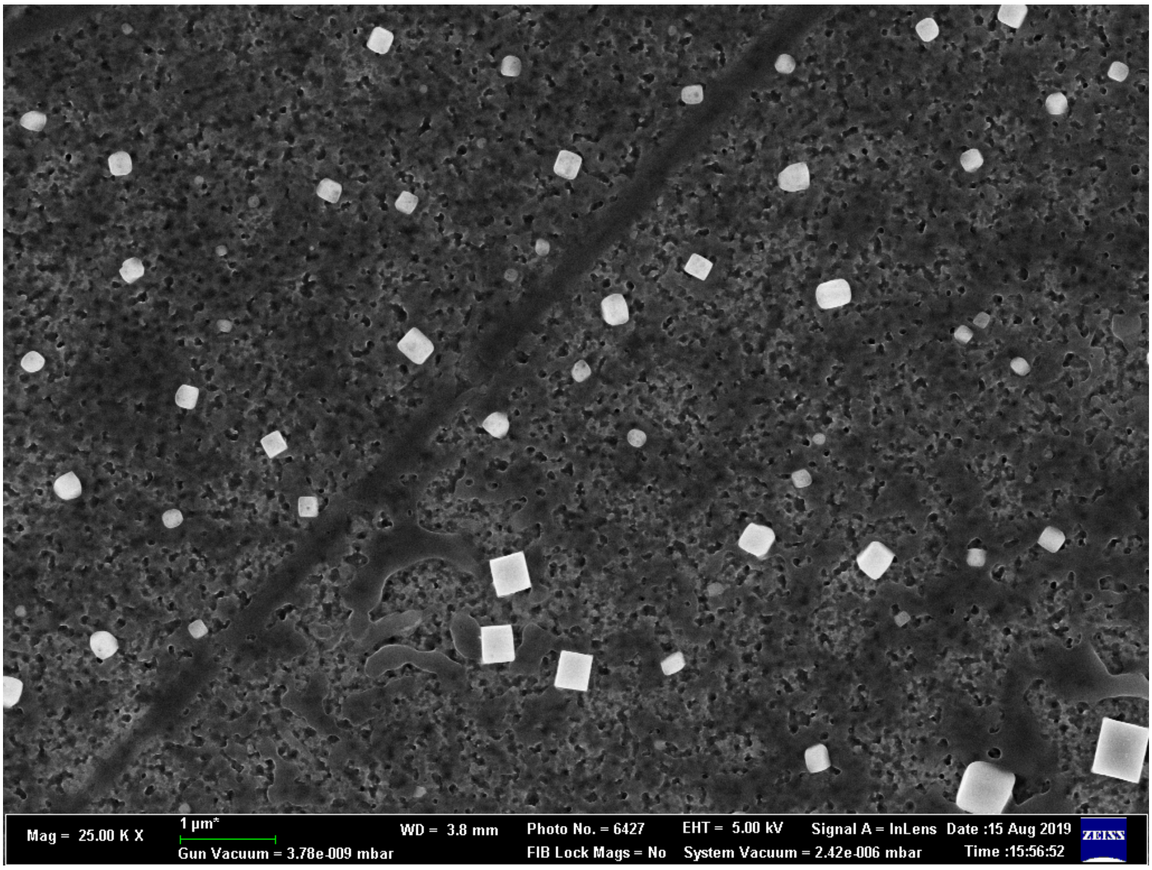

Figure S17. SEM image of AP[5]A associates.

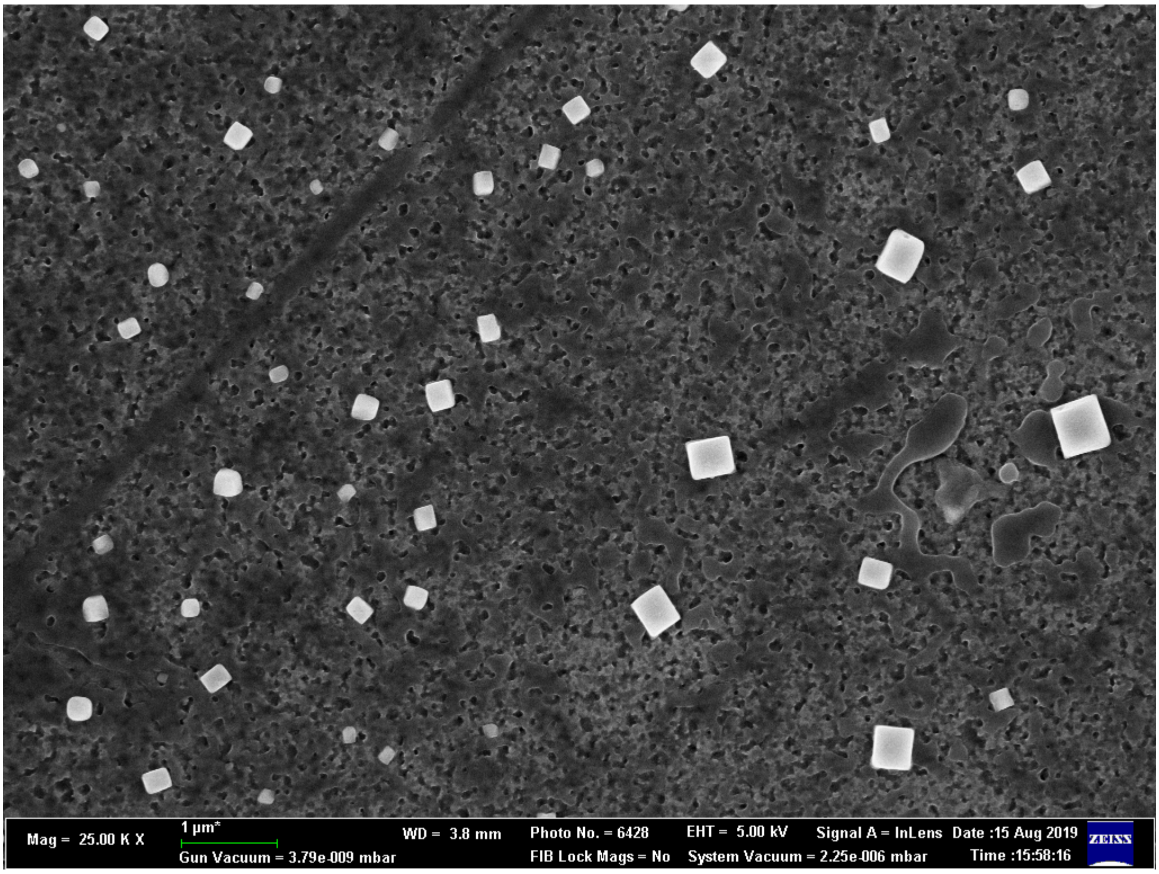

Figure S18. SEM image of AP[5]A associates.

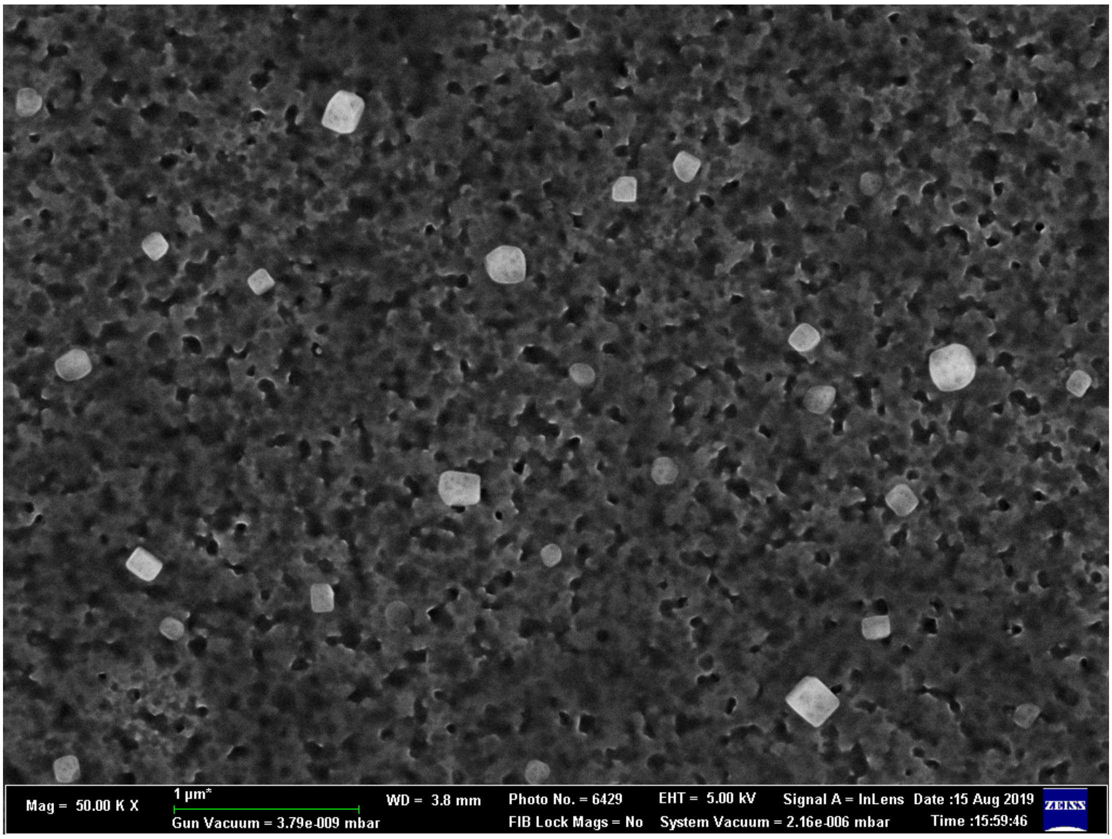

Figure S19. SEM image of AP[5]A associates.

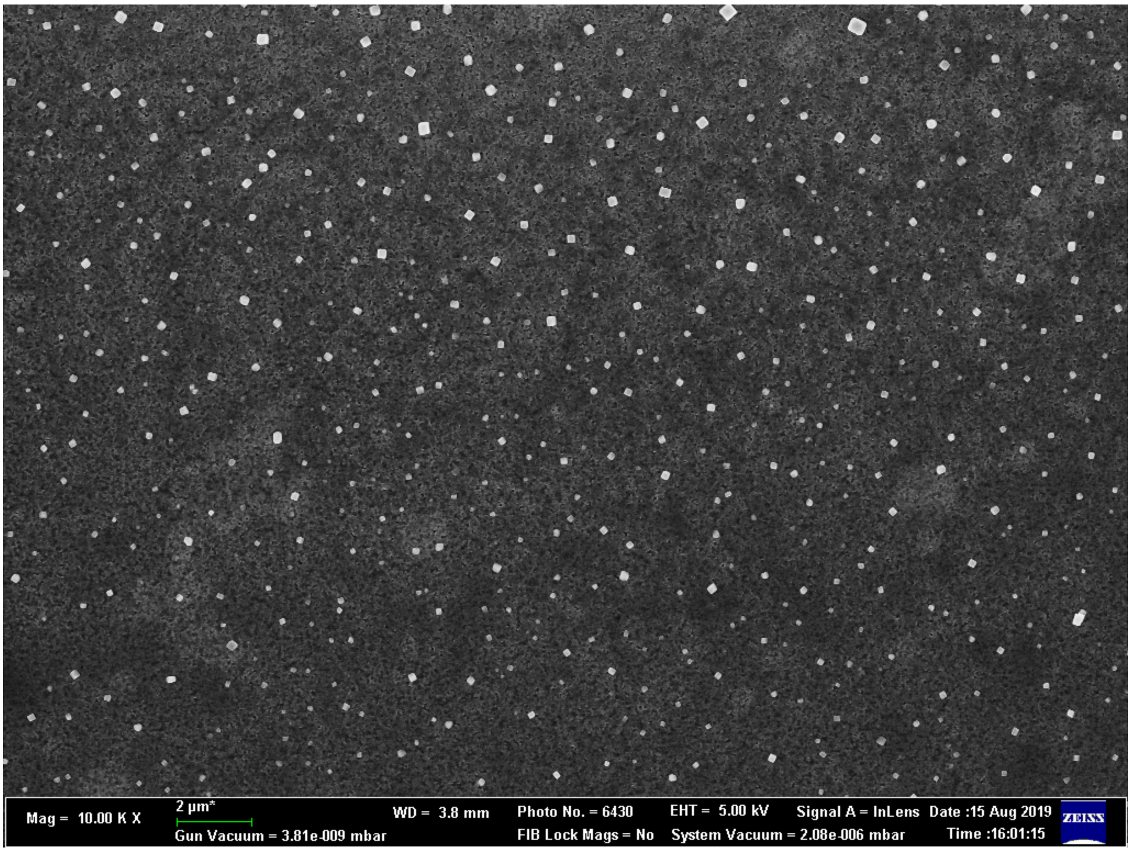

Figure S20. SEM image of AP[5]A associates.

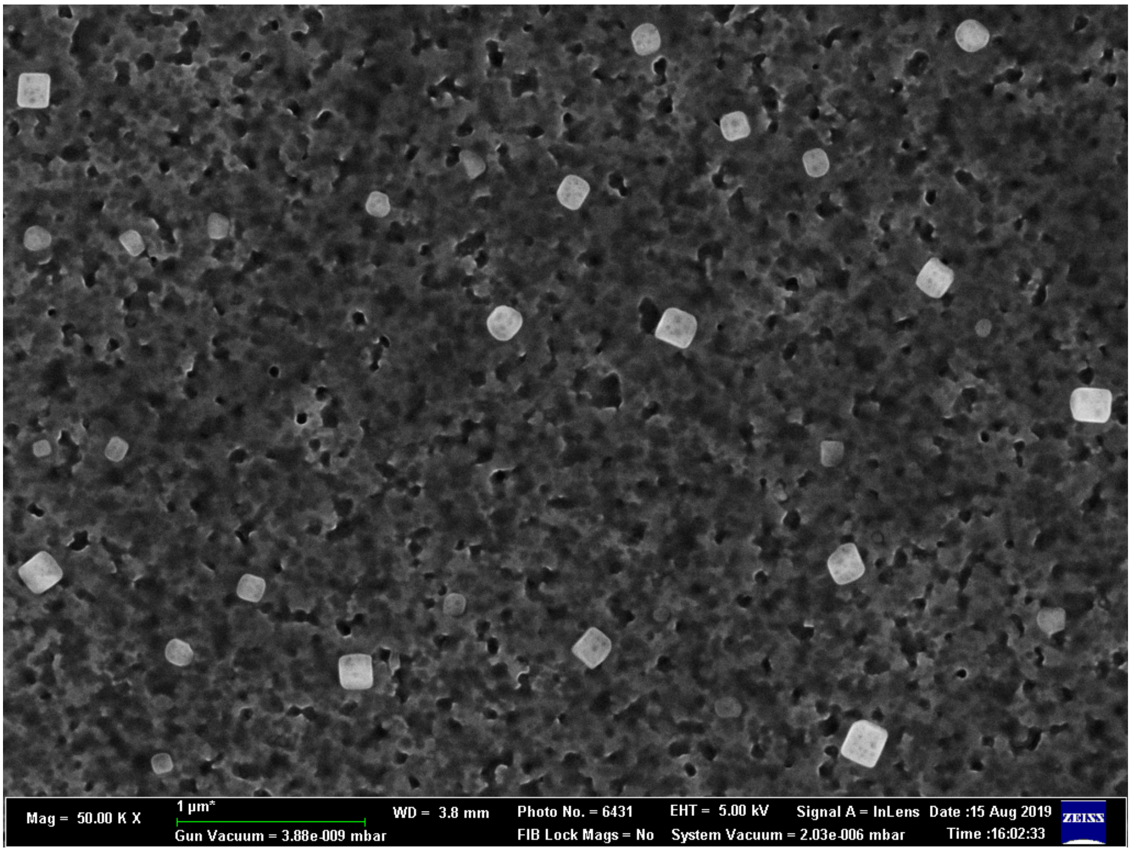

Figure S21. SEM image of AP[5]A associates.

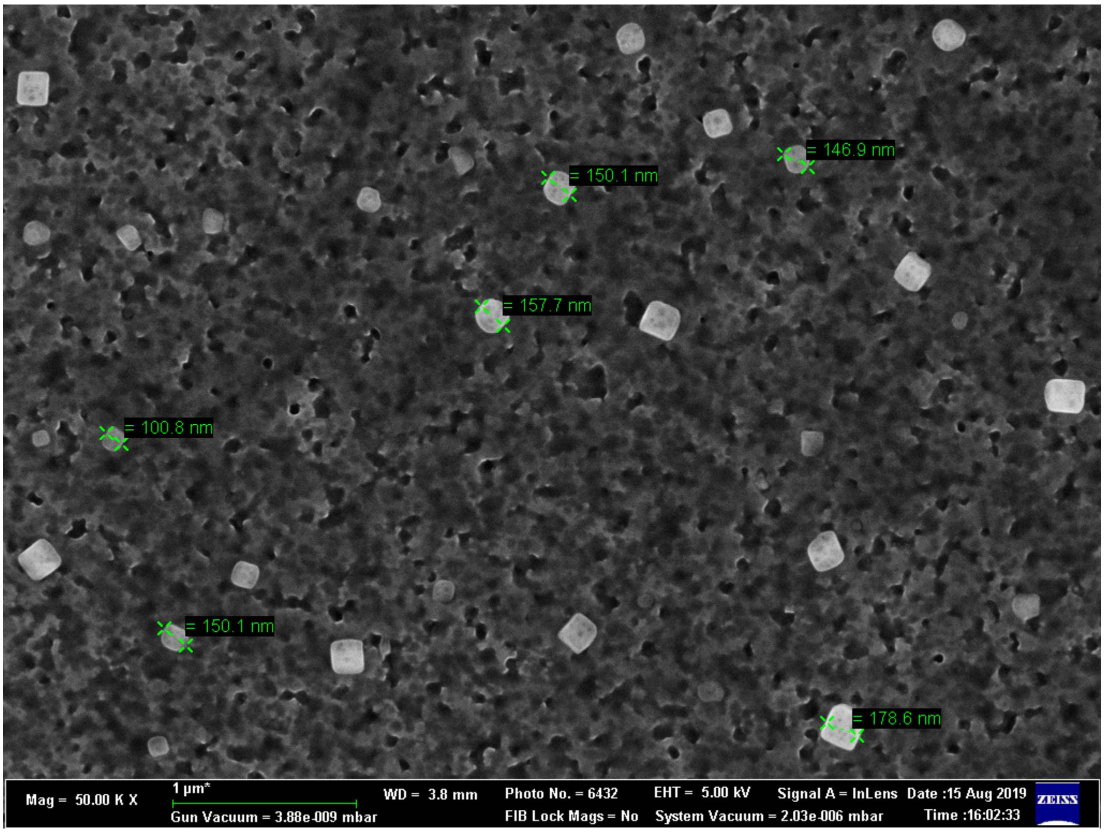

Figure S22. SEM image of AP[5]A associates.

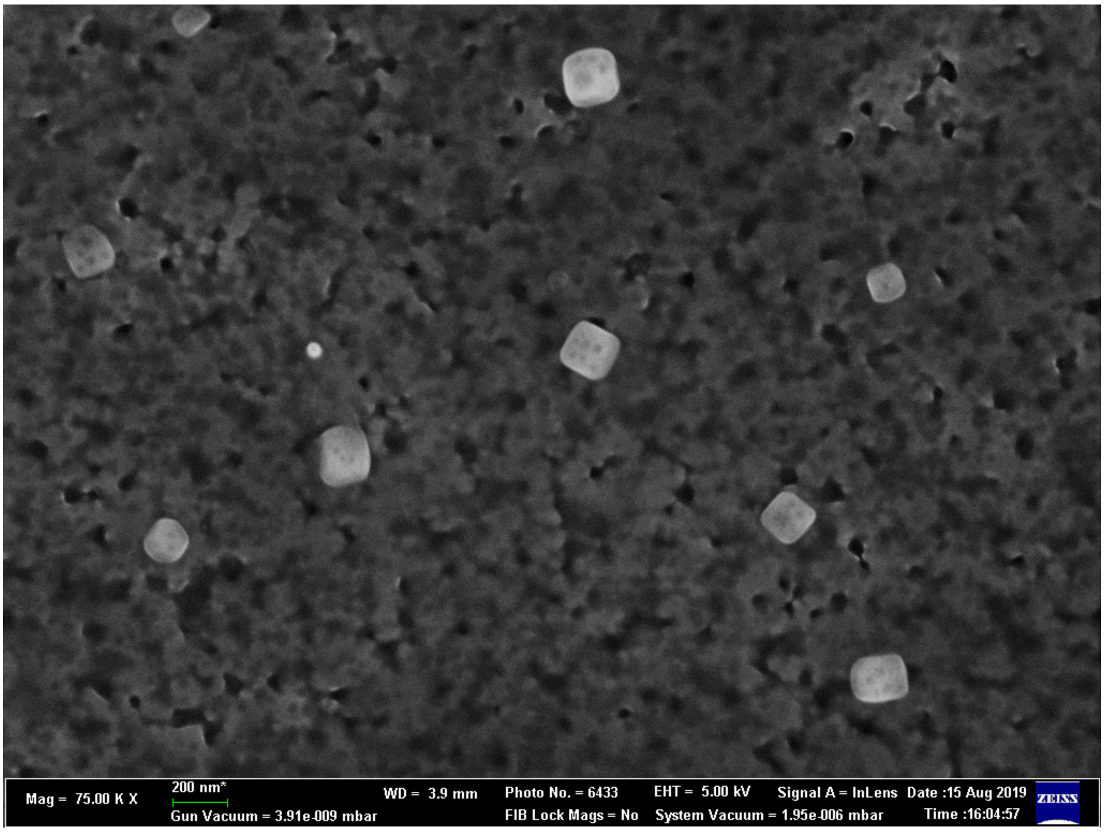

Figure S23. SEM image of AP[5]A associates.

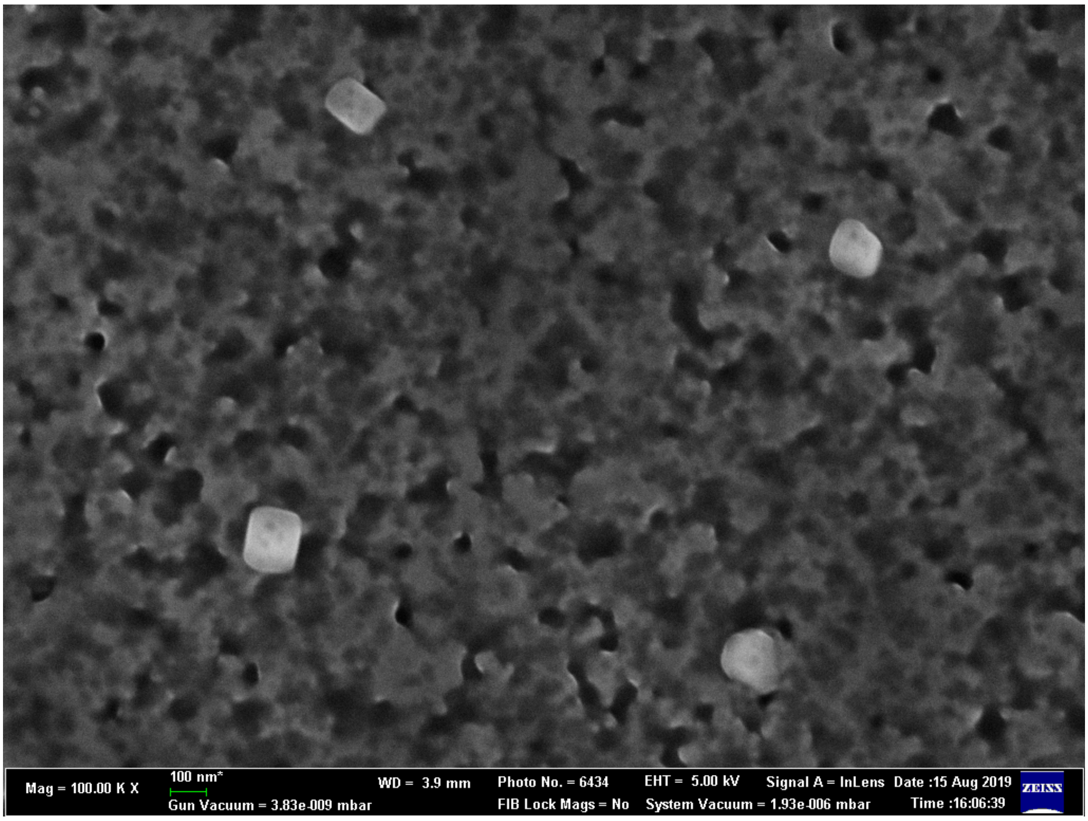

Figure S24. SEM image of AP[5]A associates.

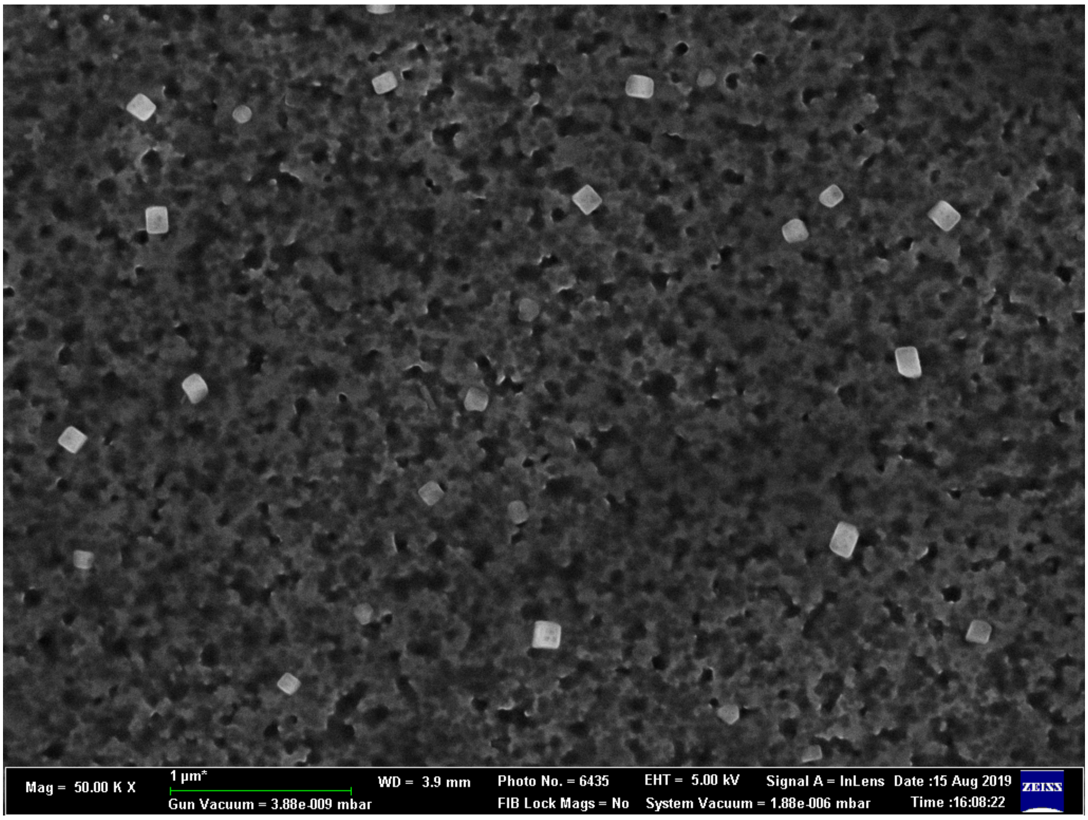

Figure S25. SEM image of AP[5]A associates.

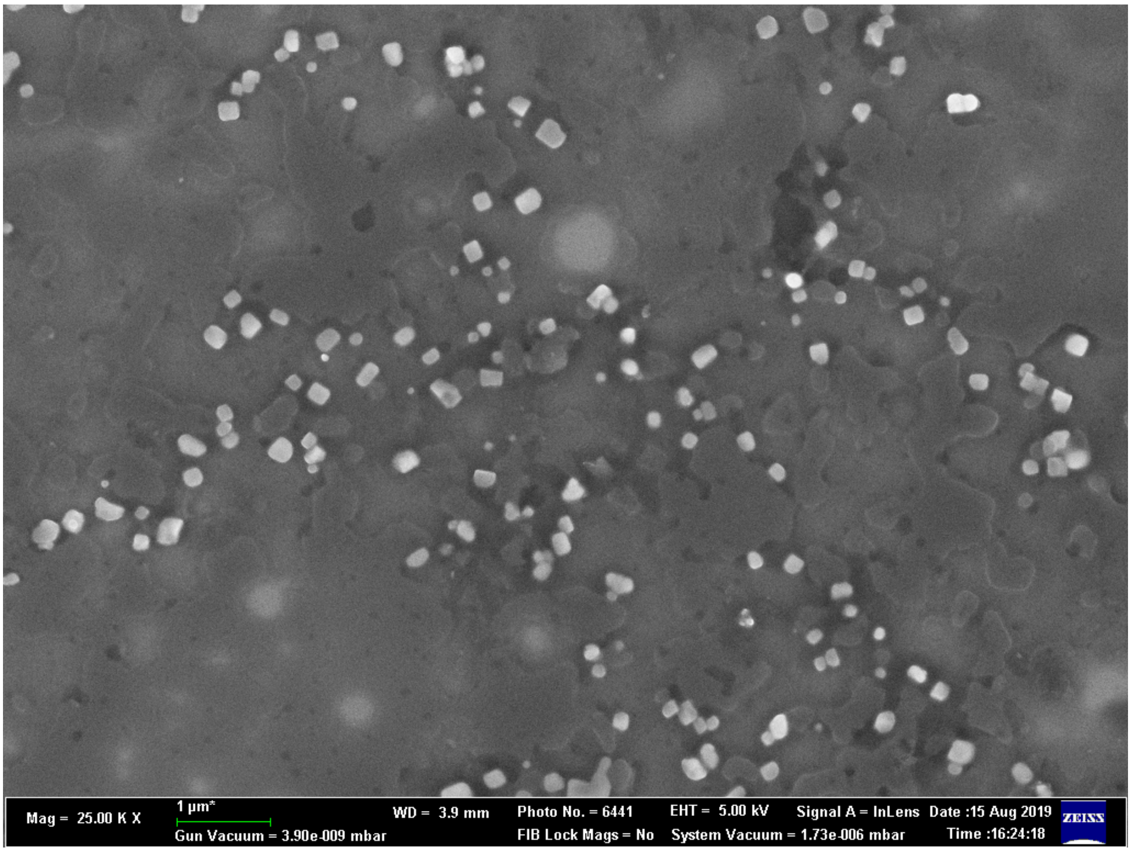

Figure S26. SEM image of AP[5]A/CT-DNA polyplex.

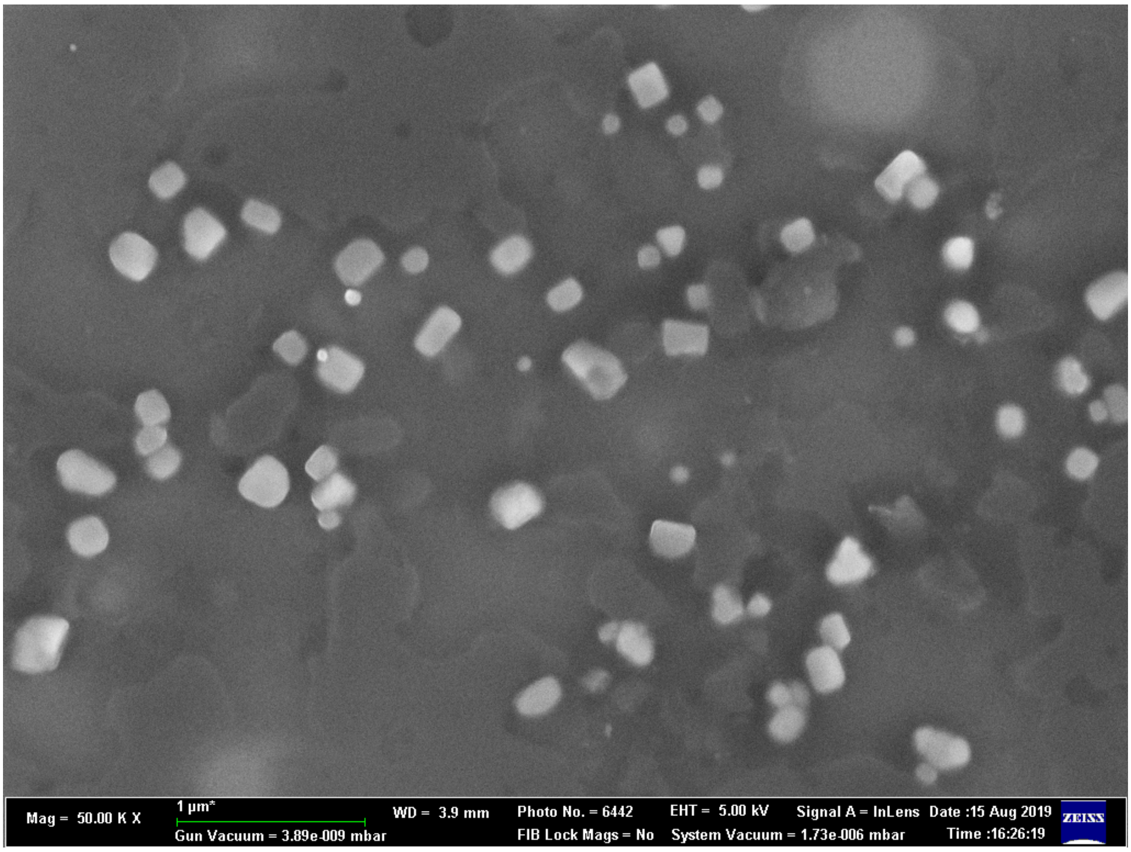

Figure S27. SEM image of AP[5]A/CT-DNA polyplex.

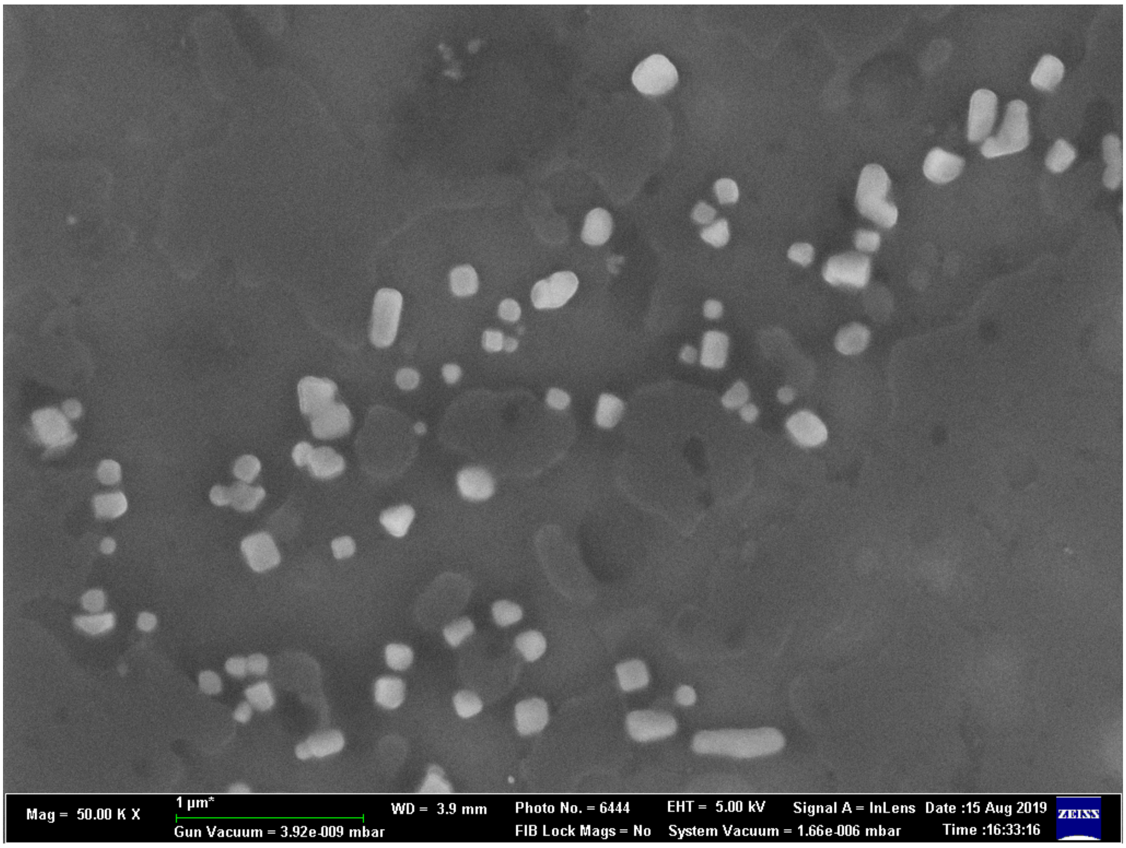

Figure S28. SEM image of AP[5]A/CT-DNA polyplex.

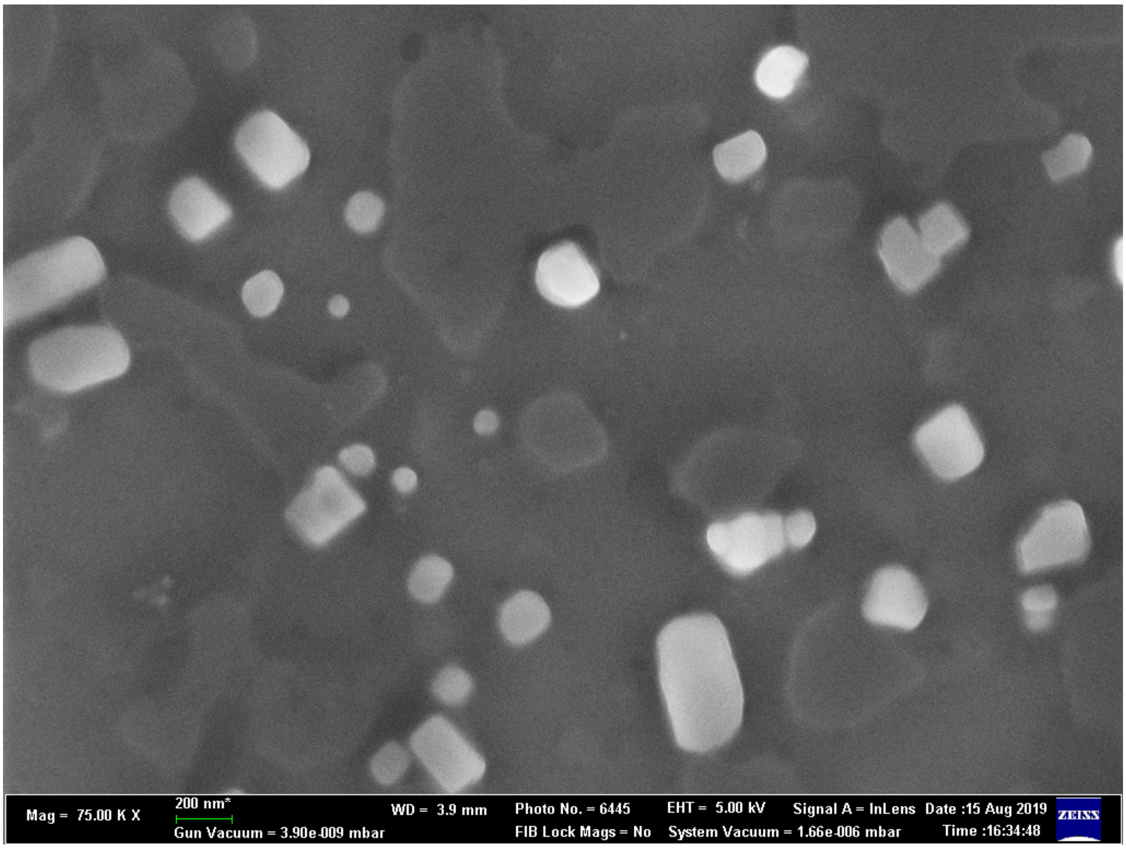

Figure S29. SEM image of AP[5]A/CT-DNA polyplex.

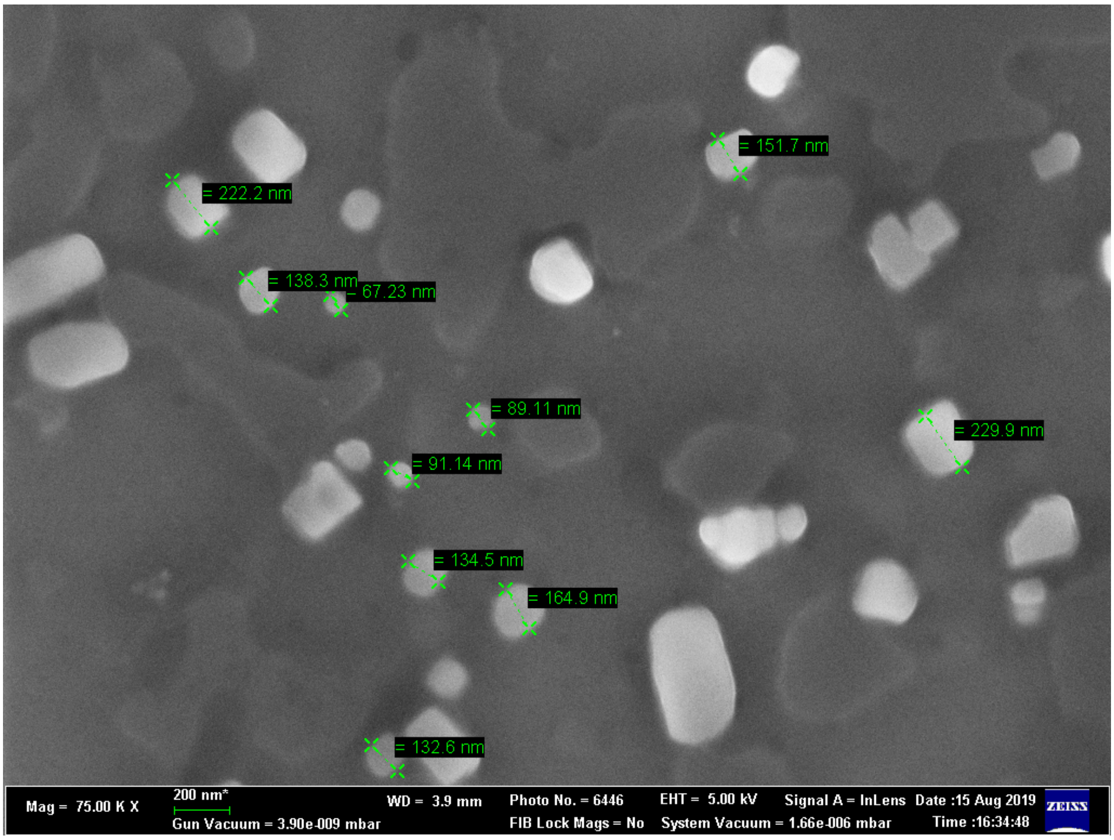

Figure S30. SEM image of AP[5]A/CT-DNA polyplex.

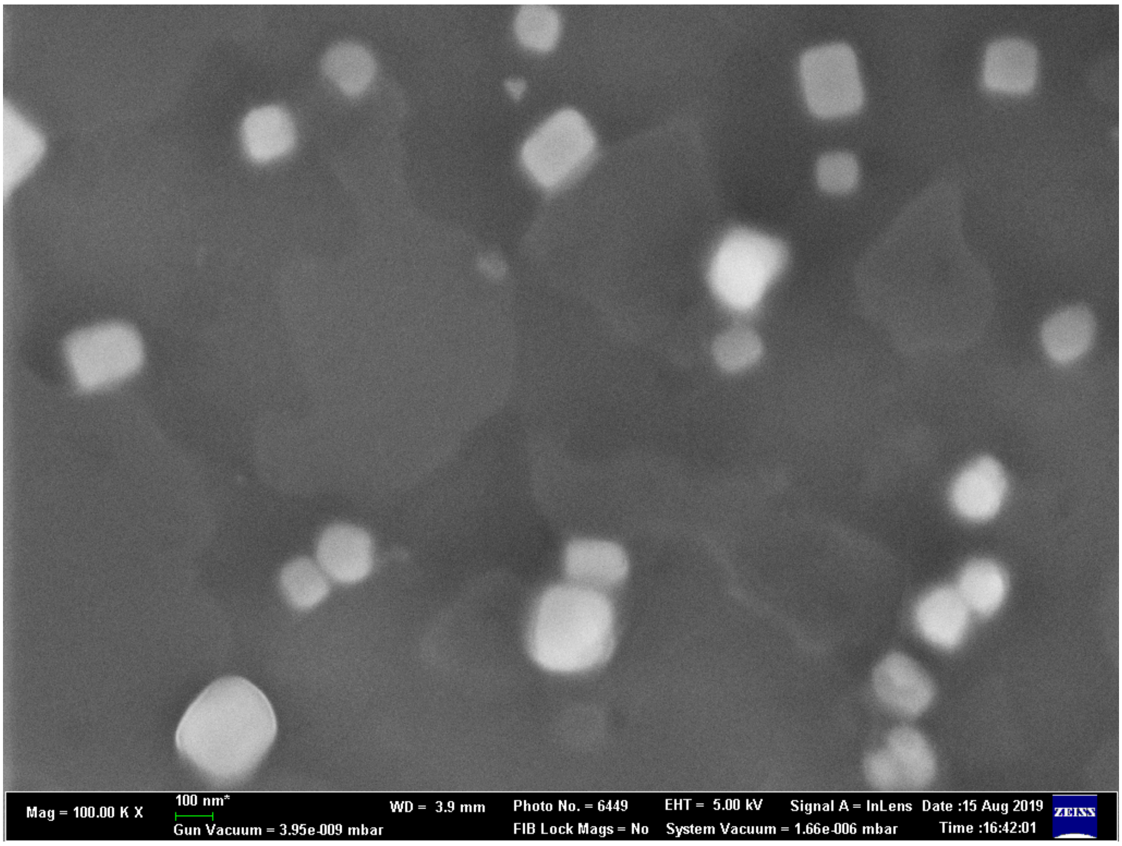

Figure S31. SEM image of AP[5]A/CT-DNA polyplex.

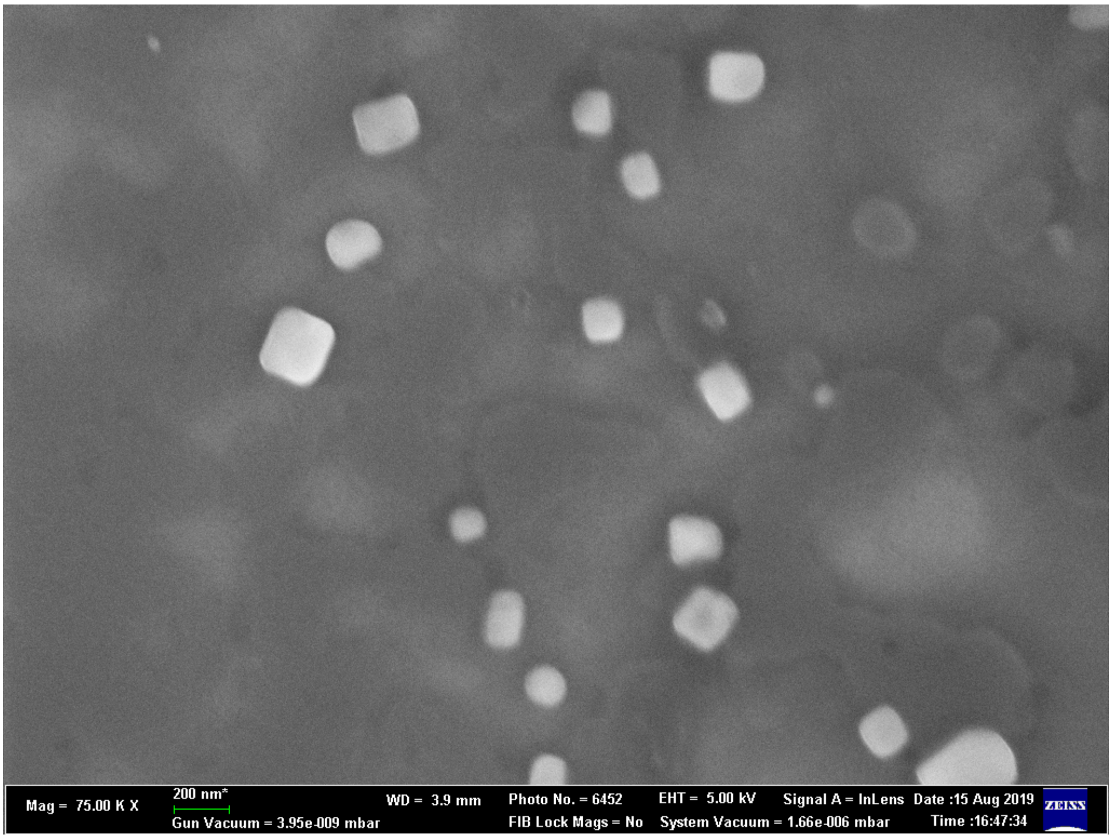

Figure S32. SEM image of AP[5]A/CT-DNA polyplex.

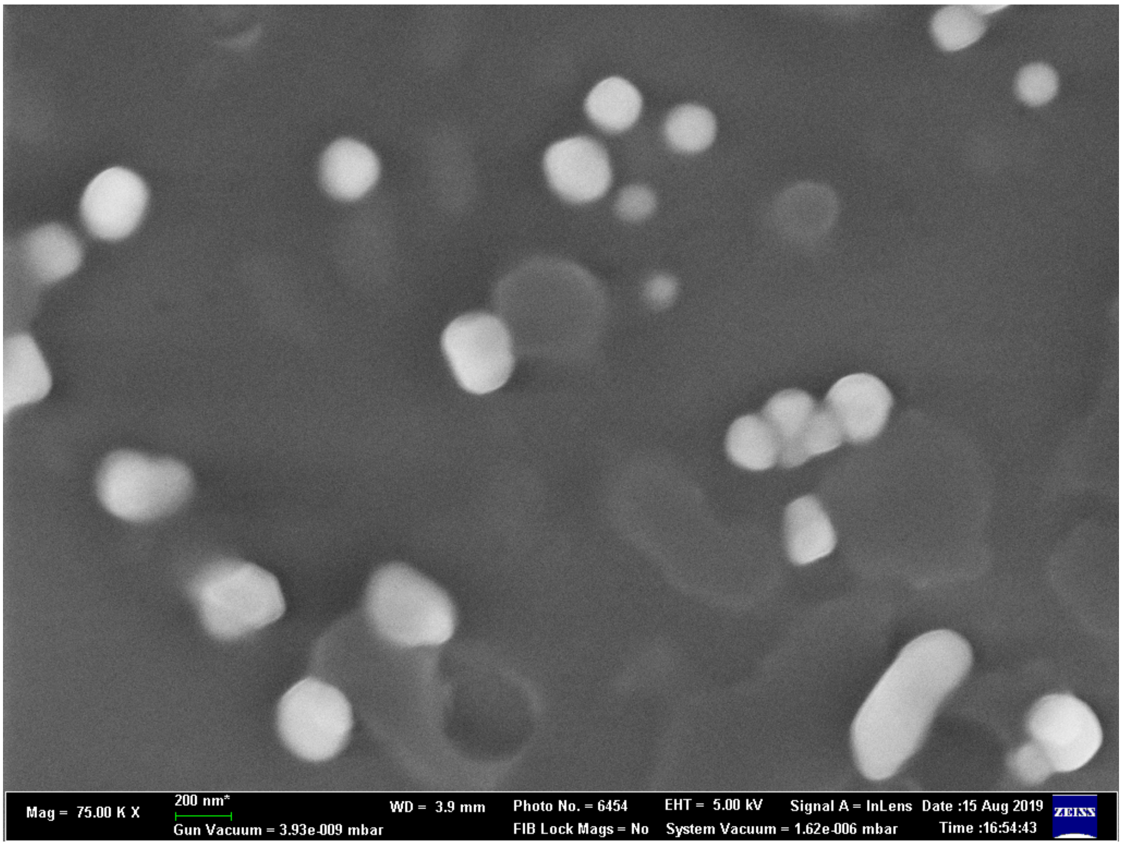

Figure S33. SEM image of AP[5]A/CT-DNA polyplex.

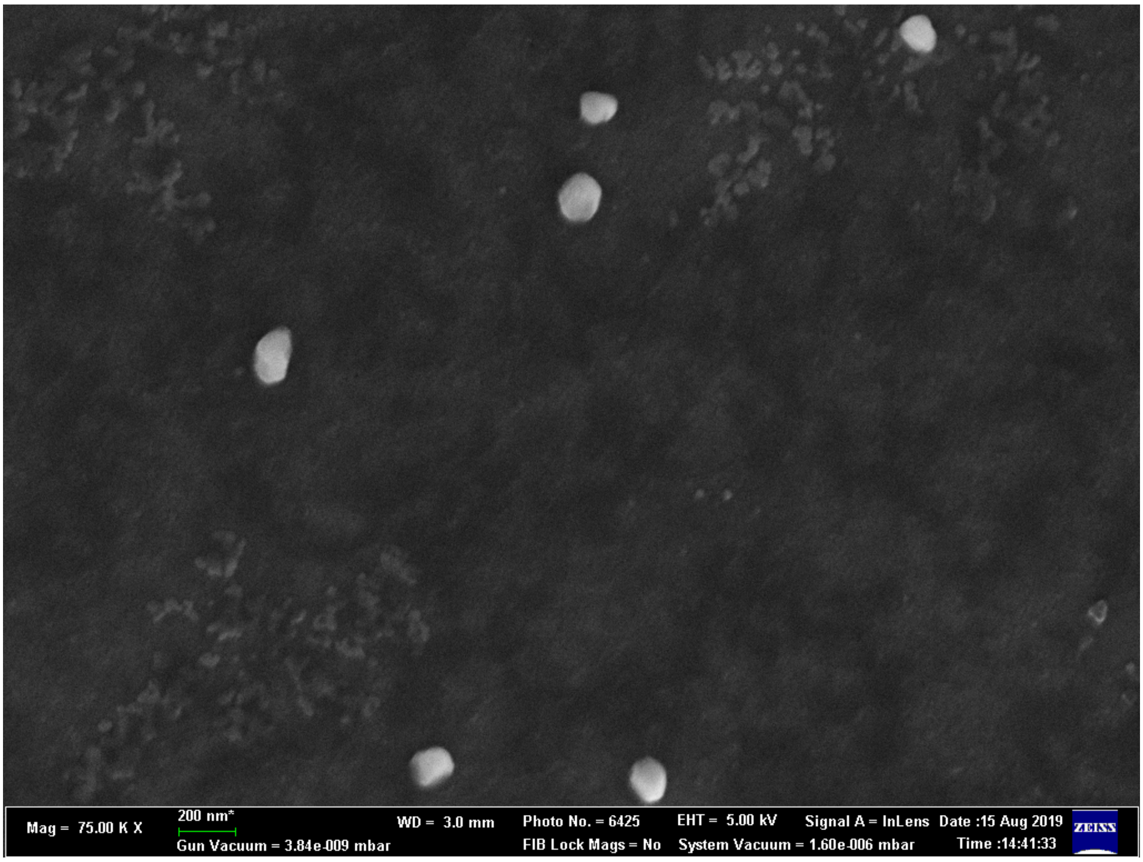

Figure S34. SEM image of co-interpolyelectrolyte associate AP[5]A/STC[4]A.

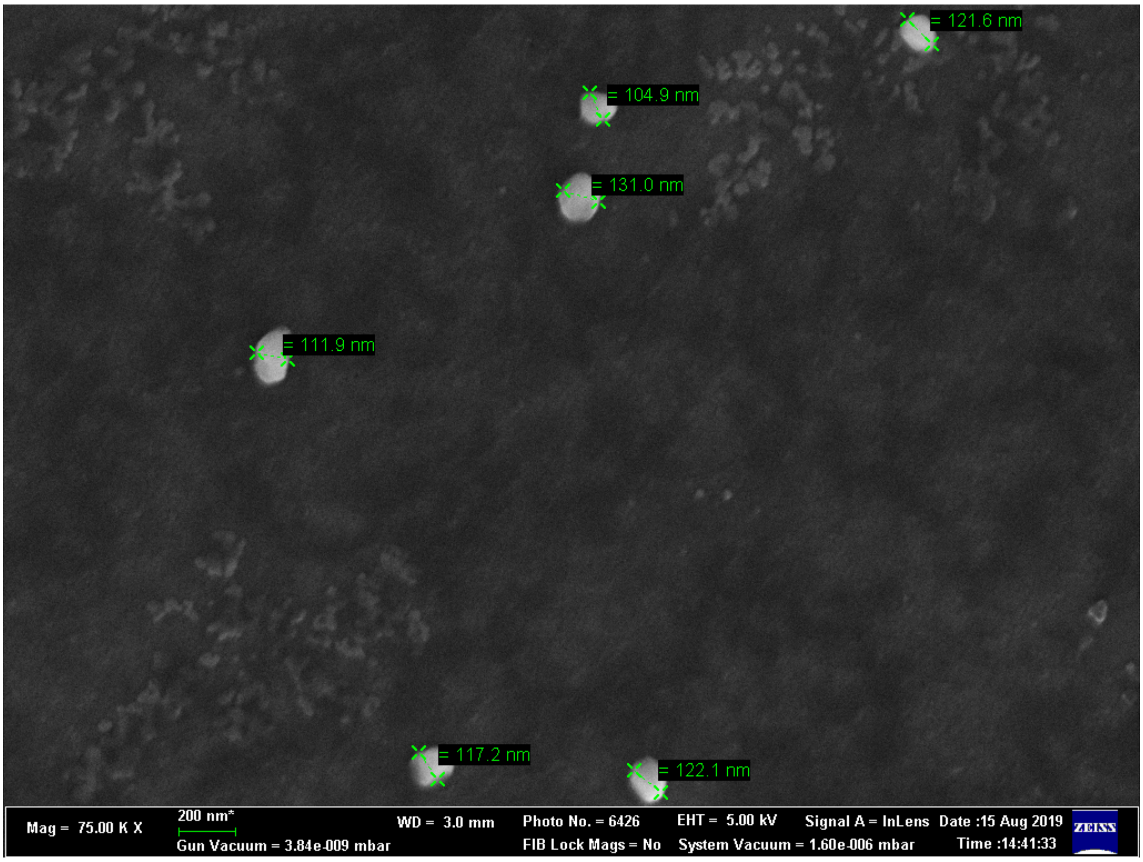

Figure S35. SEM image of co-interpolyelectrolyte associate AP[5]A/STC[4]A.

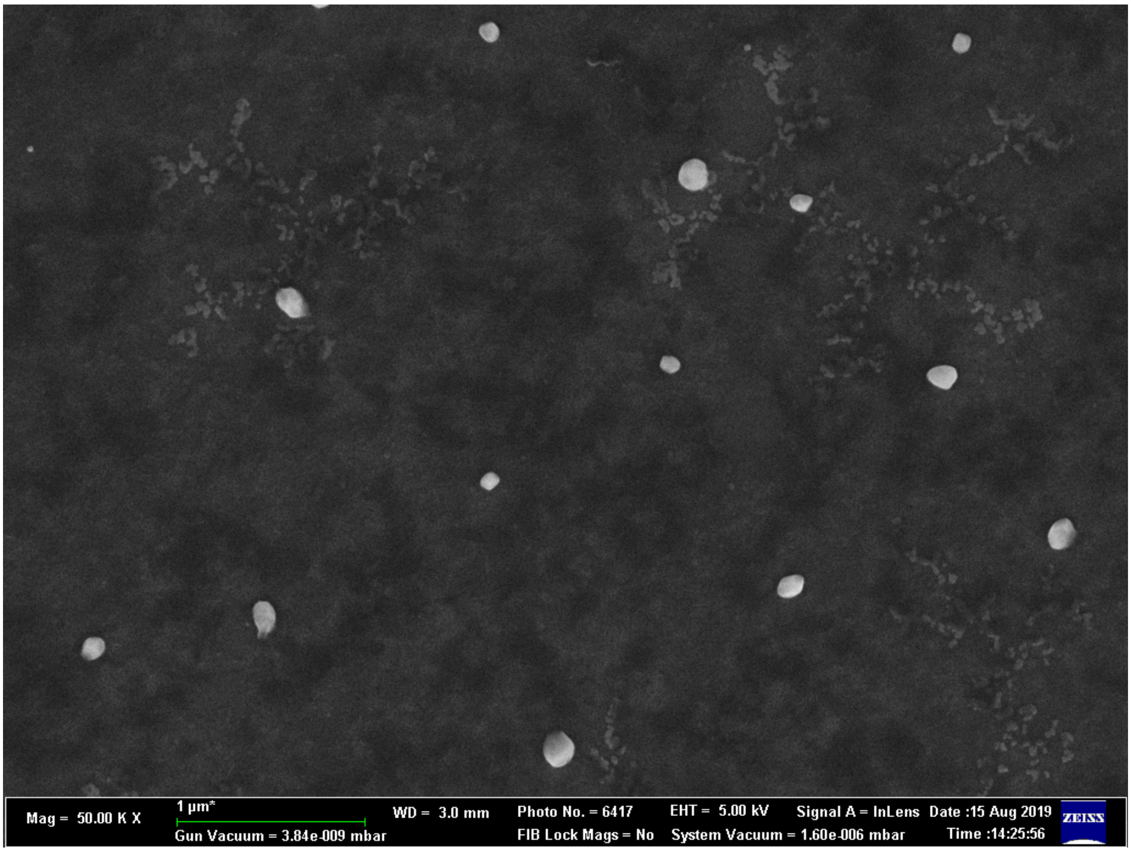

Figure S36. SEM image of co-interpolyelectrolyte associate AP[5]A/STC[4]A.

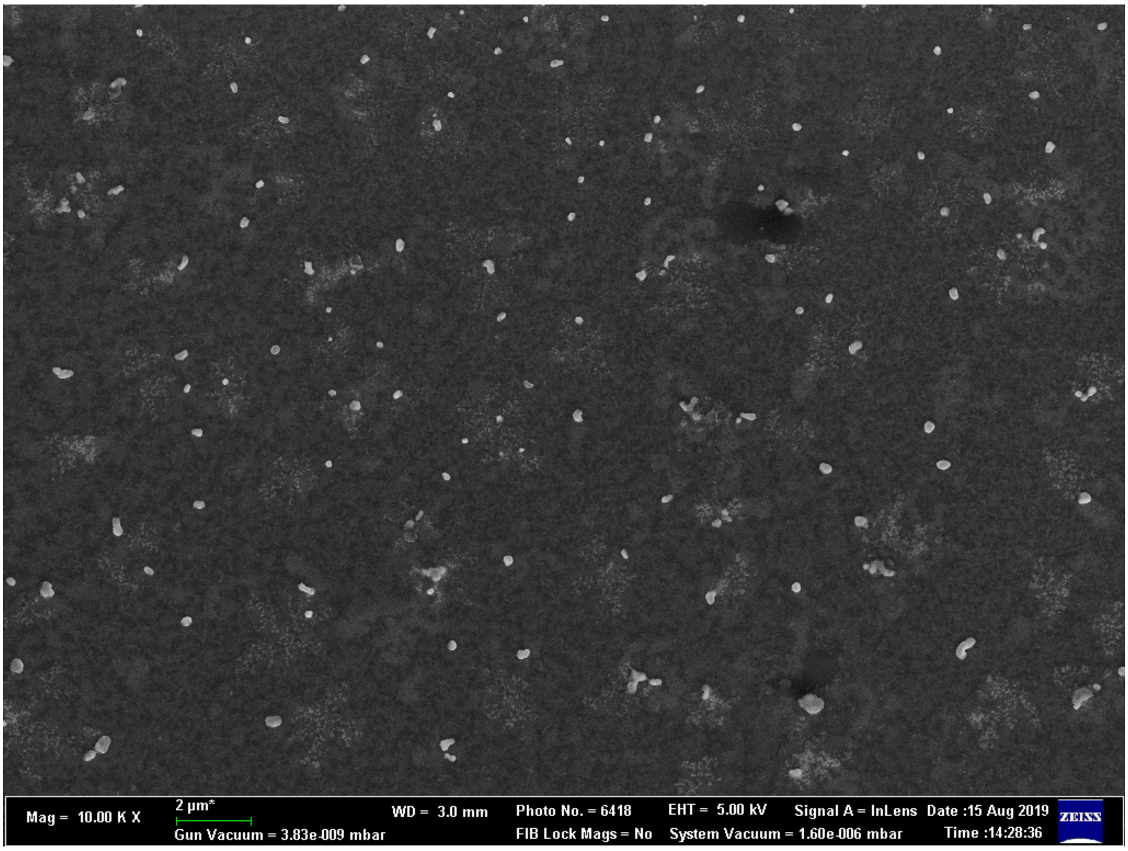

Figure S37. SEM image of co-interpolyelectrolyte associate AP[5]A/STC[4]A.

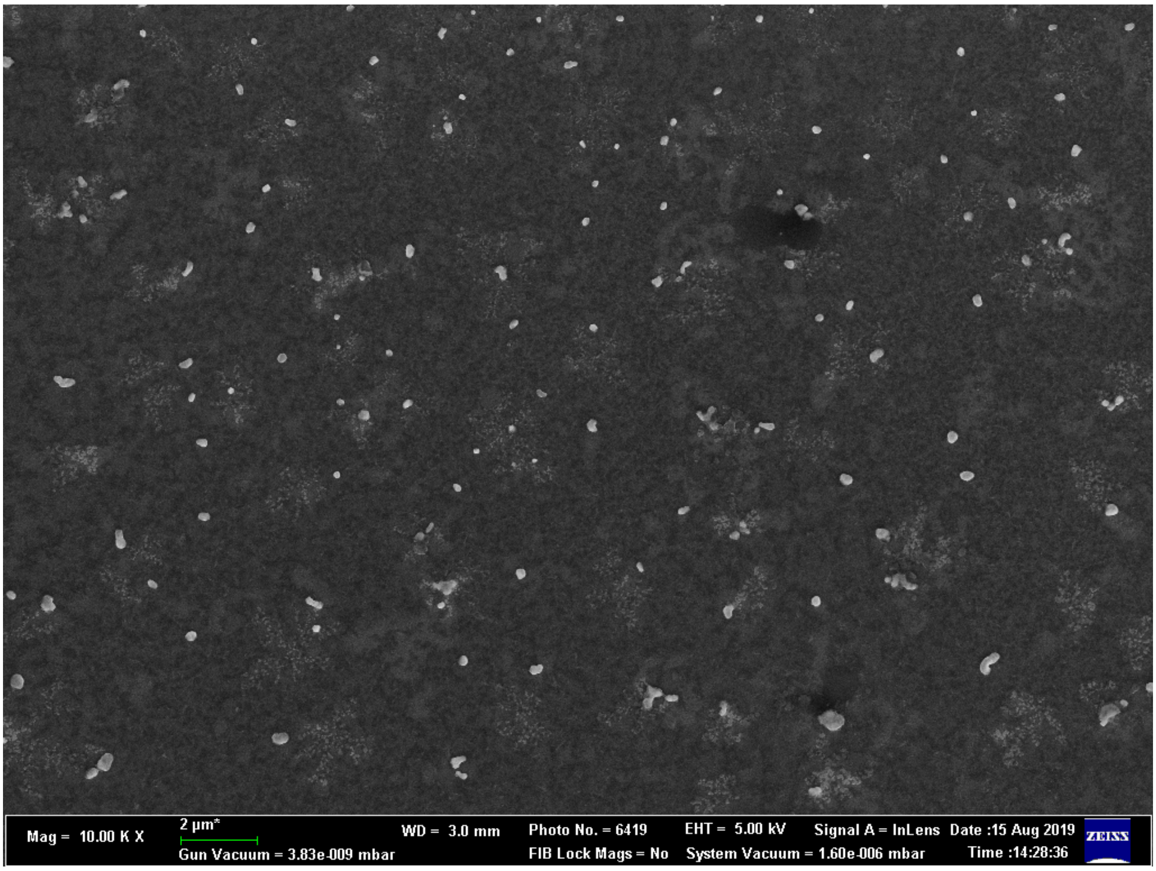

Figure S38. SEM image of co-interpolyelectrolyte associate AP[5]A/STC[4]A.

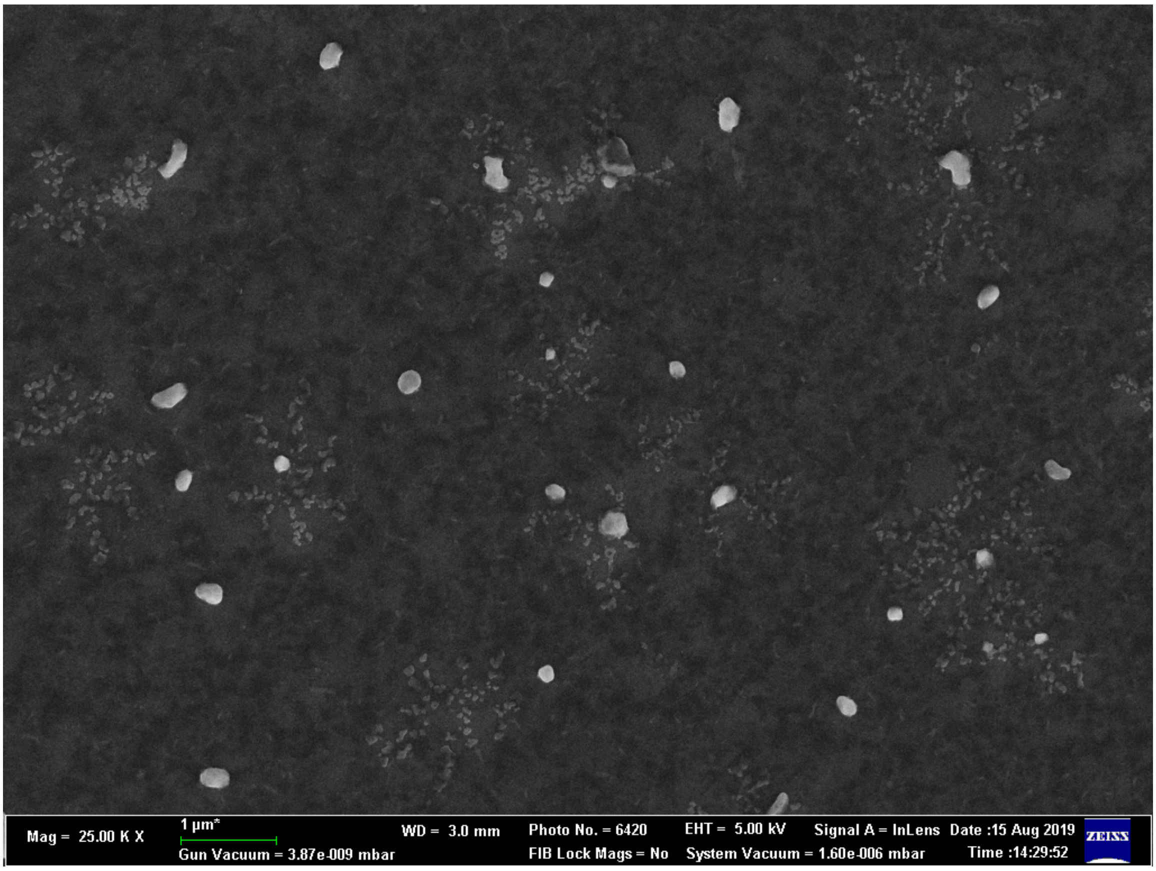

Figure S39. SEM image of co-interpolyelectrolyte associate AP[5]A/STC[4]A.

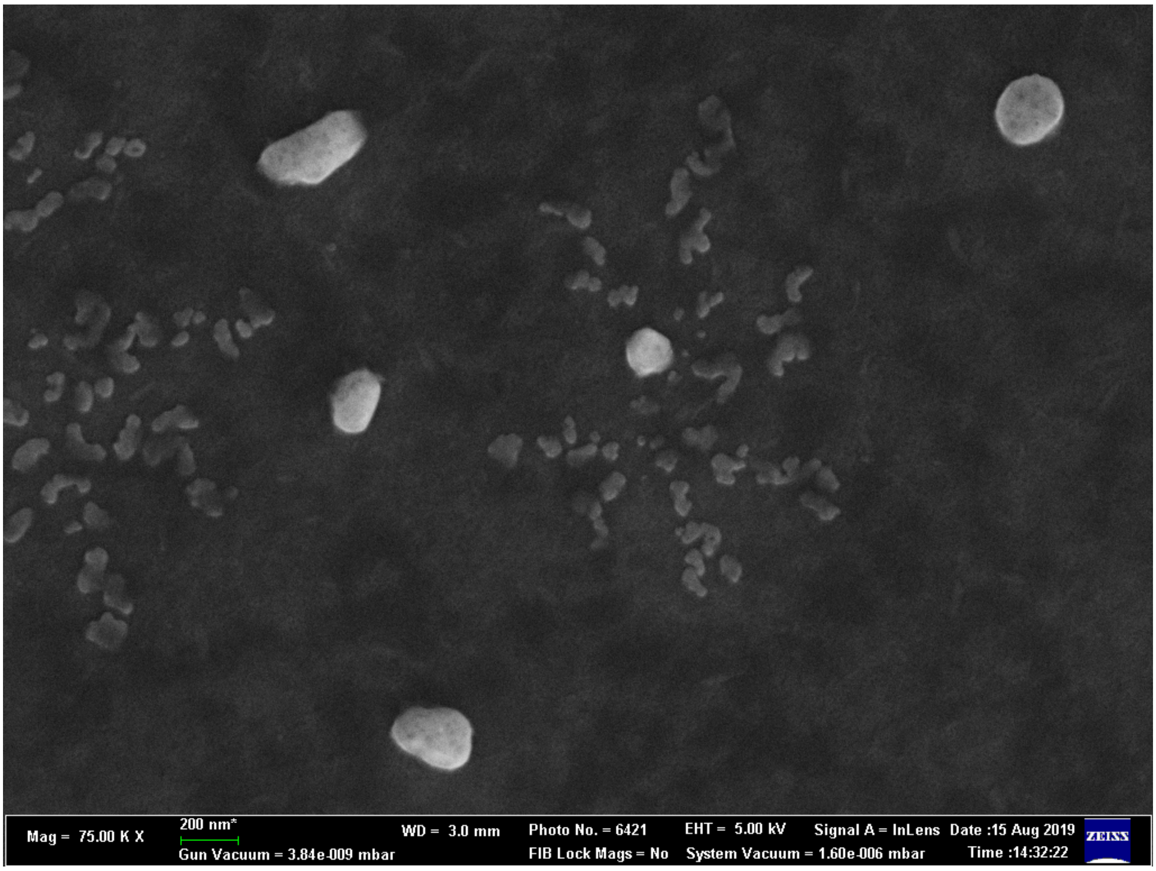

Figure S40. SEM image of co-interpolyelectrolyte associate AP[5]A/STC[4]A.

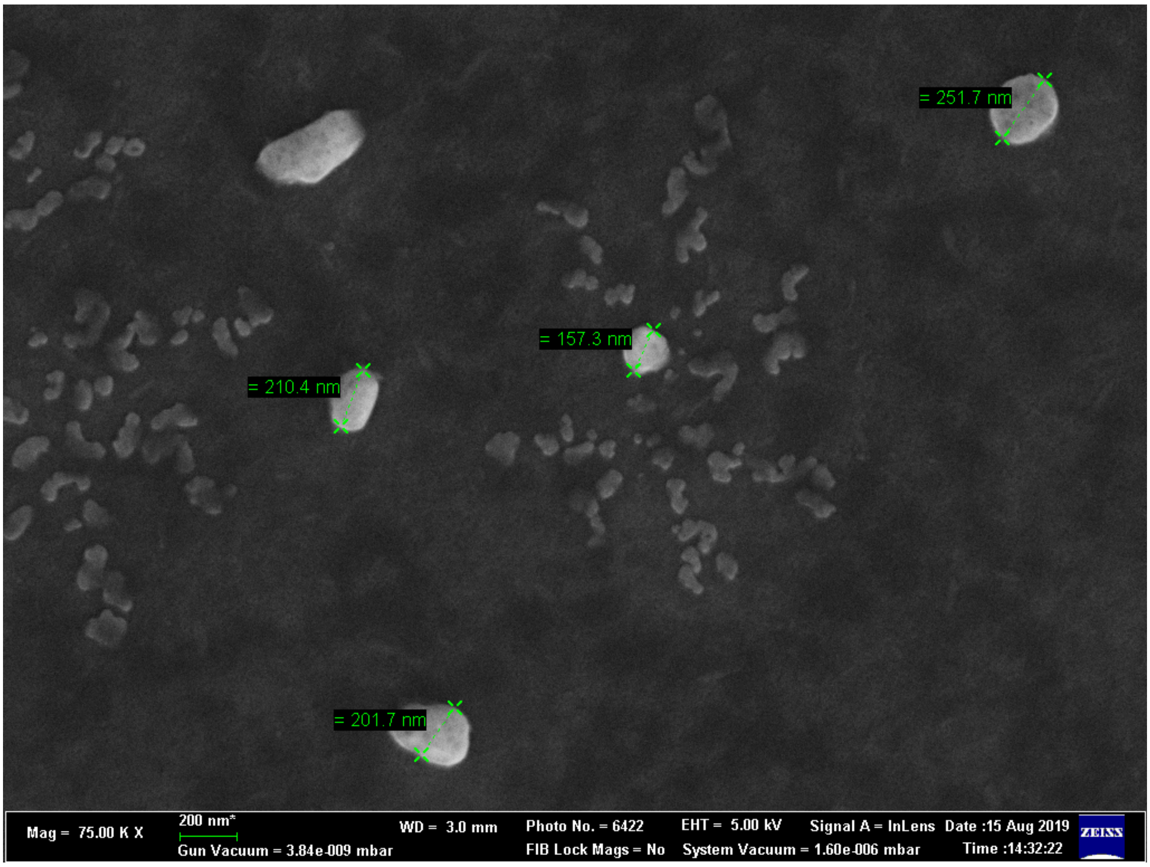

Figure S41. SEM image of co-interpolyelectrolyte associate AP[5]A/STC[4]A.

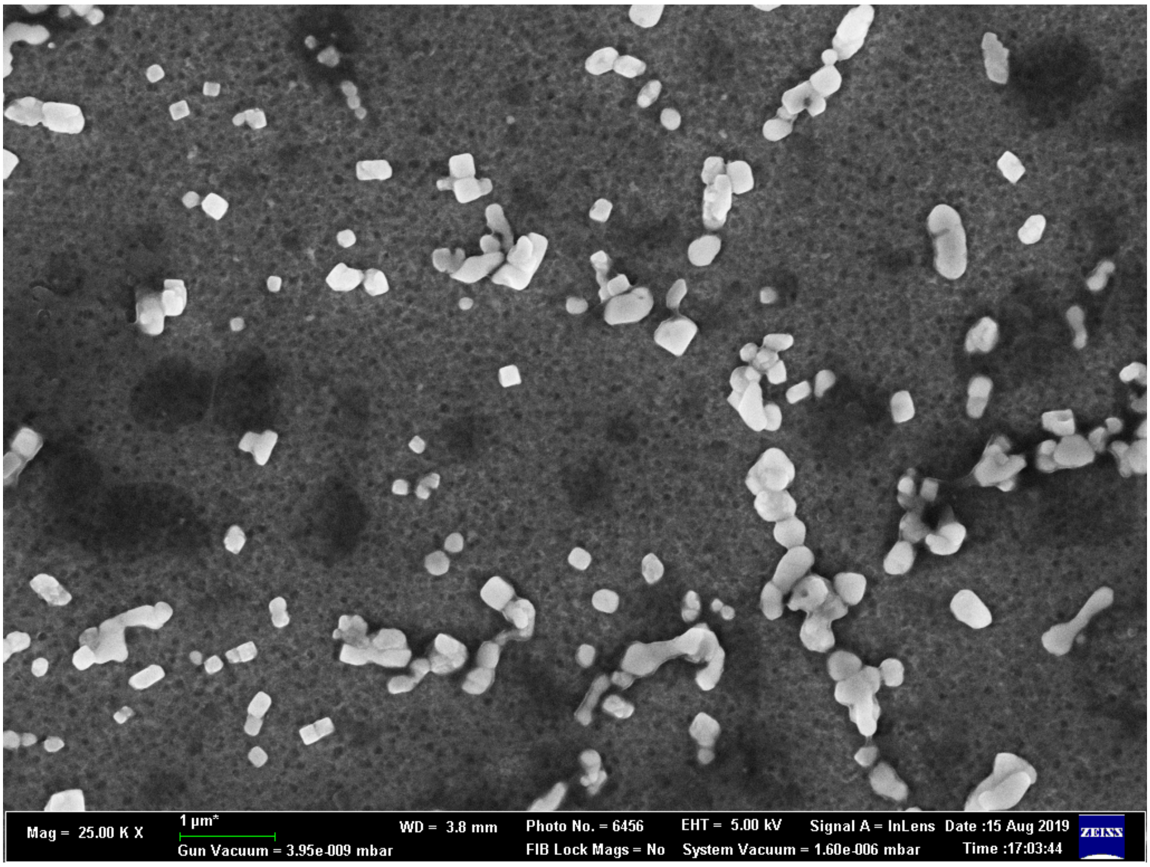

Figure S42. SEM image of AP[5]A/STC[4]A/CT-DNA micelleplexes.

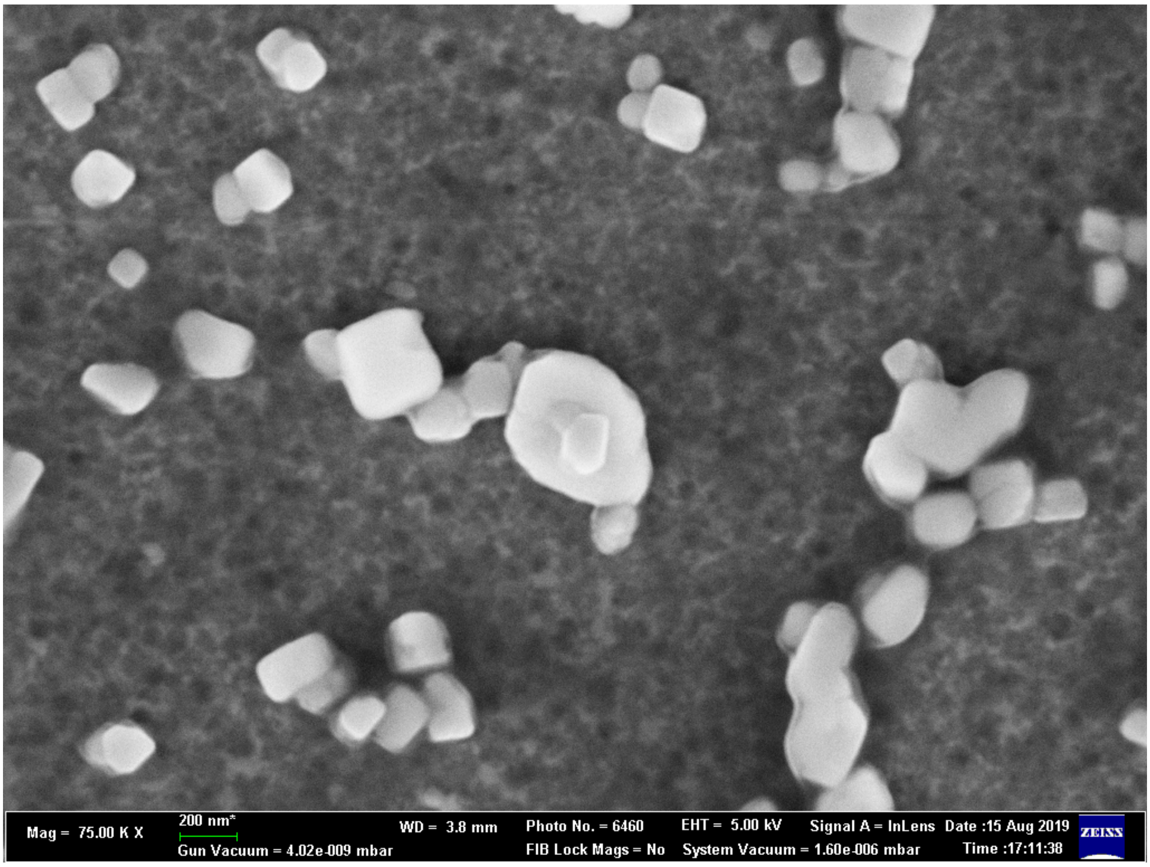

Figure S43. SEM image of AP[5]A/STC[4]A/CT-DNA micelleplexes.

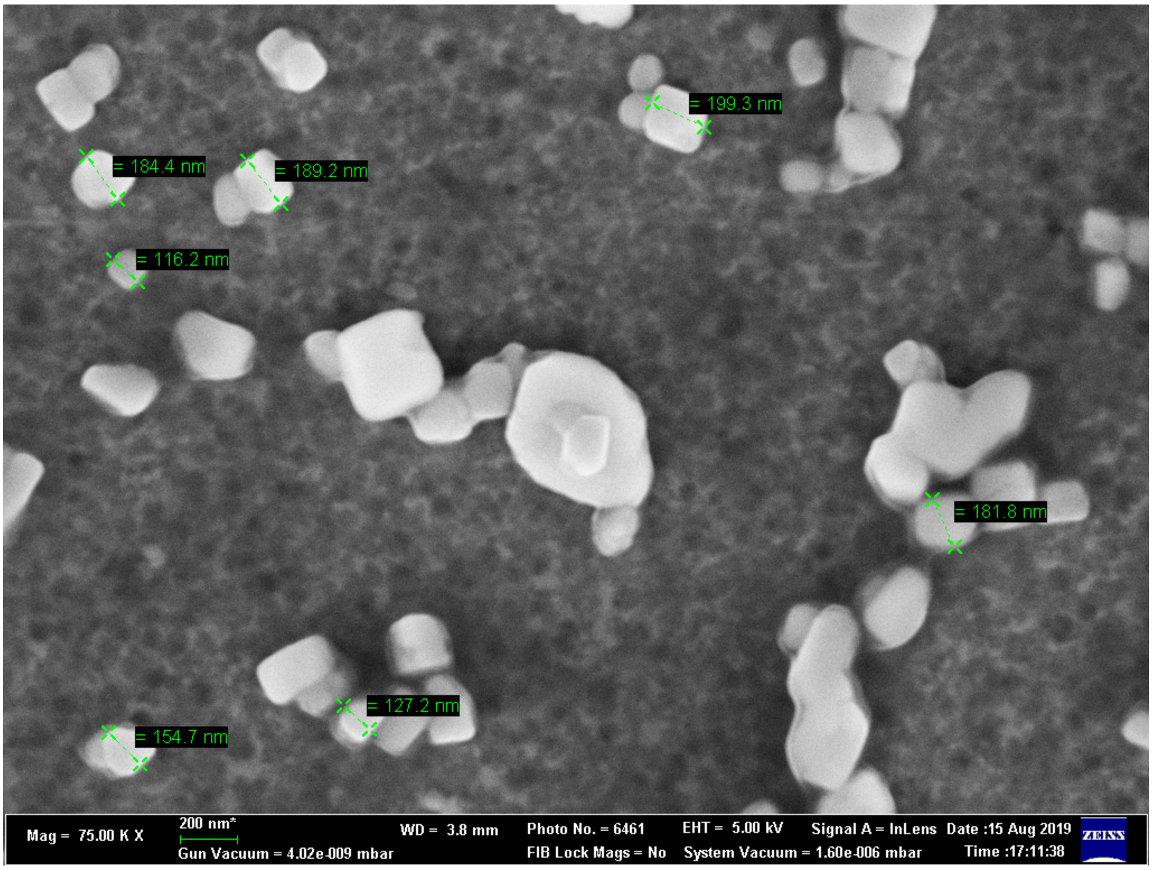

Figure S44. SEM image of AP[5]A/STC[4]A/CT-DNA micelleplexes.

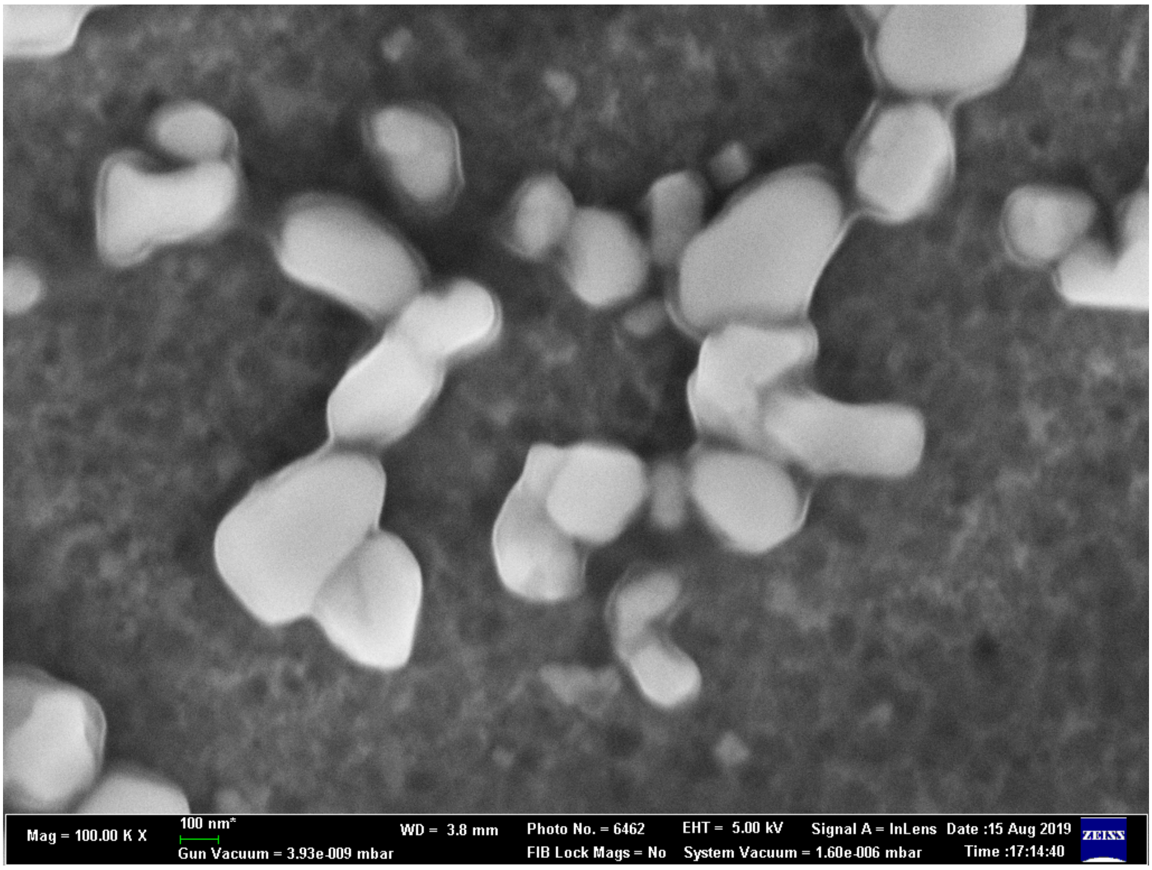

Figure S45. SEM image of AP[5]A/STC[4]A/CT-DNA micelleplexes.

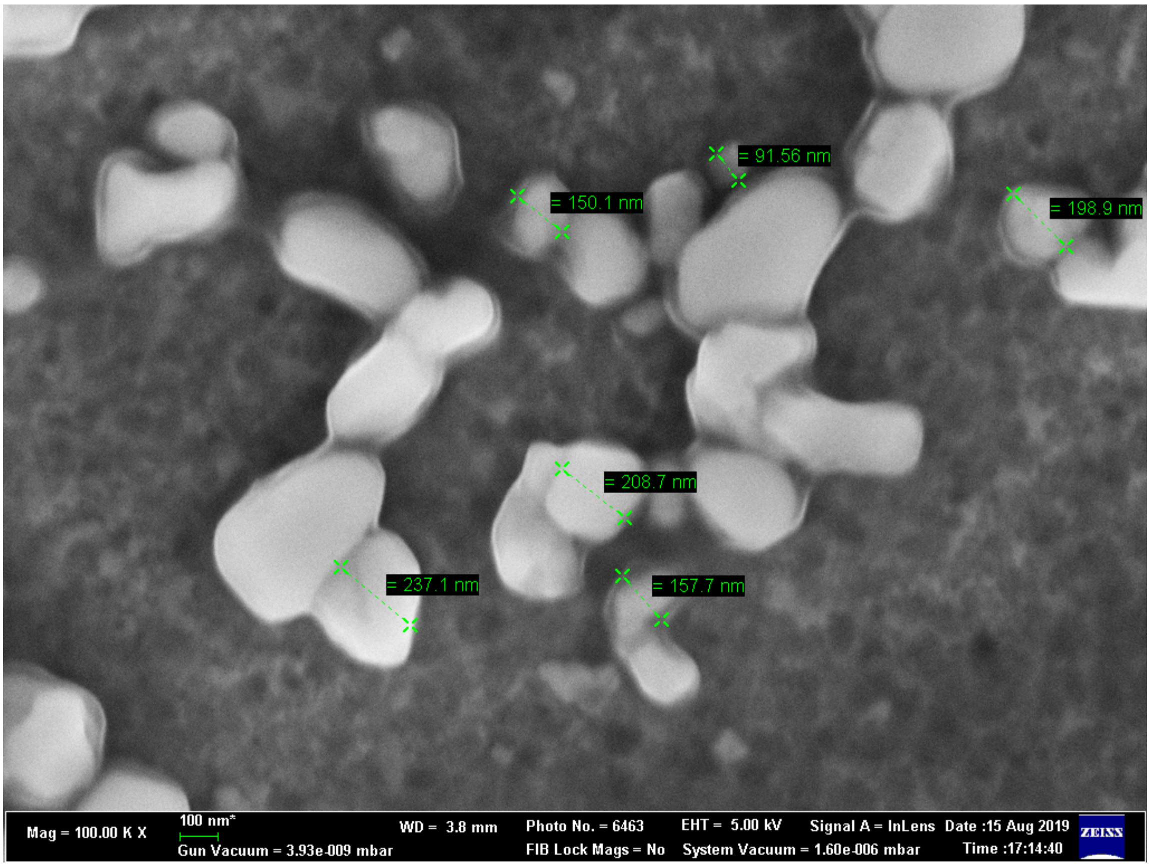

Figure S46. SEM image of AP[5]A/STC[4]A/CT-DNA micelleplexes.

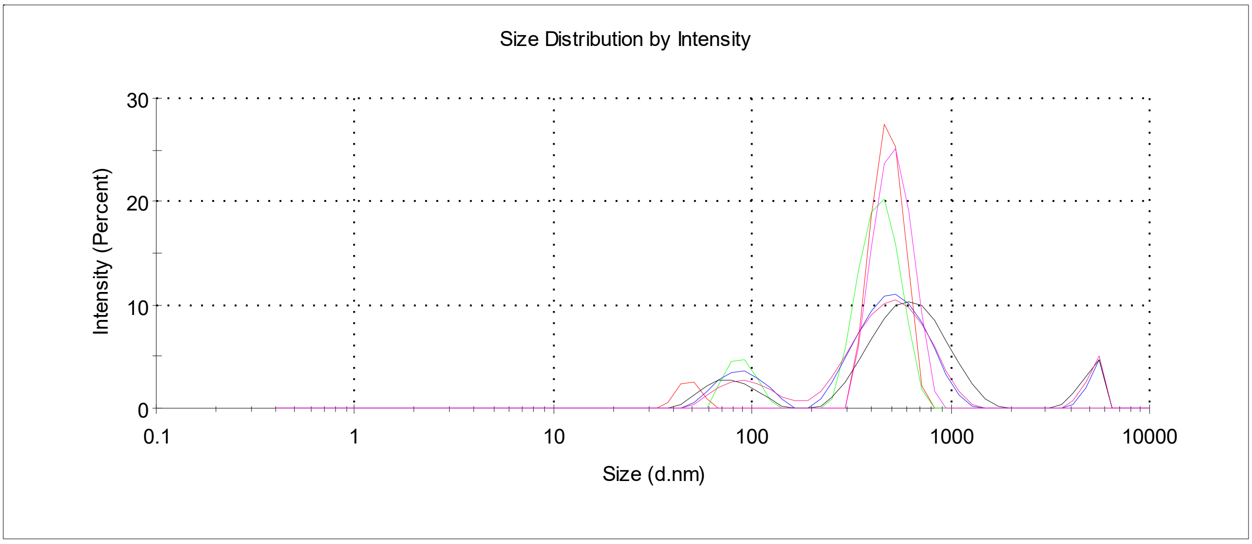

Figure S47. Size distribution of the associates AP[5]A, c = 3 × 10^{-4} M. Each line in figure is one measurement from six.

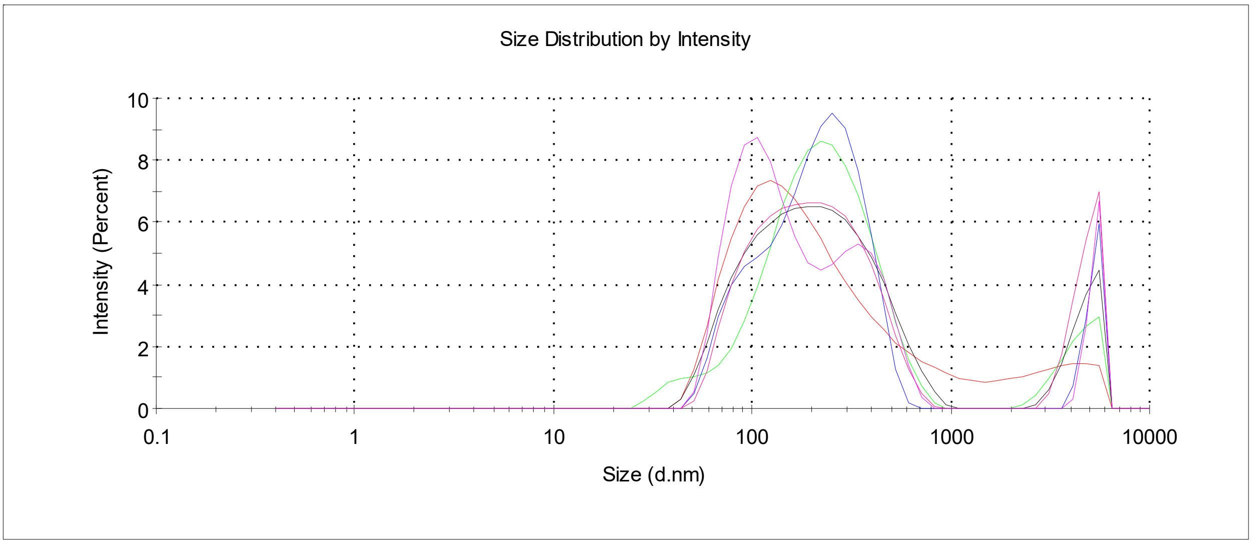

Figure S48. Size distribution of the associates AP[5]A, c = 3 × 10^{-5} M. Each line in figure is one measurement from six.

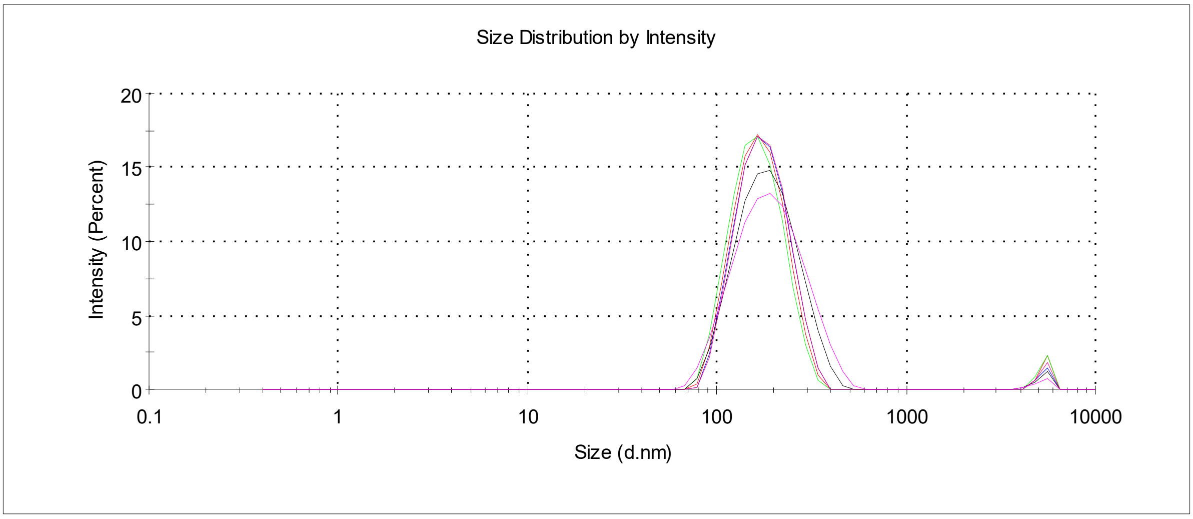

Figure S49. Size distribution of the associates AP[5]A, c = 3 × 10^{-6} M. Each line in figure is one measurement from six.

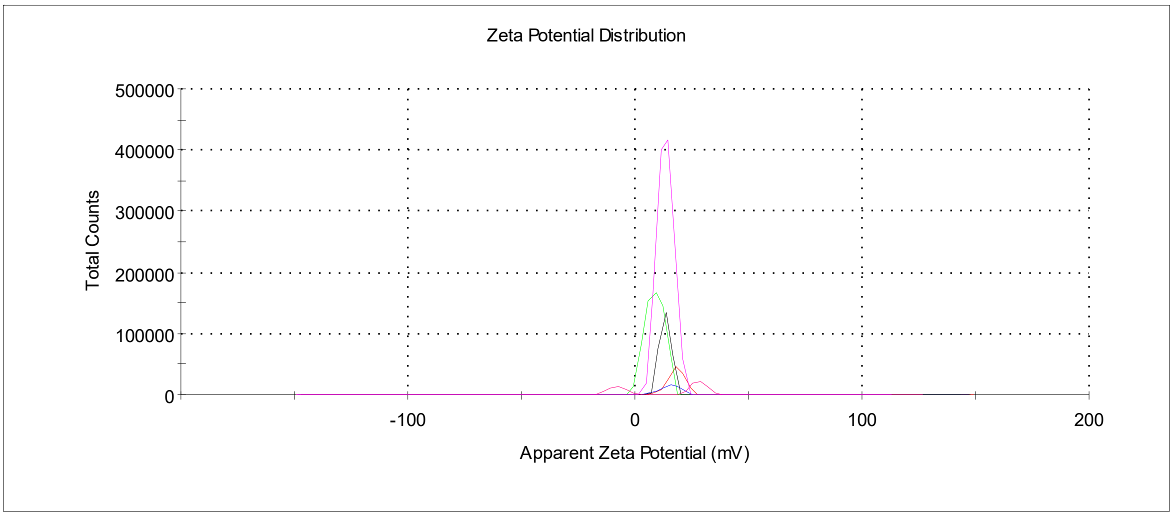

Figure S50. Zeta potential distributions of the associates AP[5]A, c = 3 × 10^{-5} M. Each line in figure is one measurement from six.

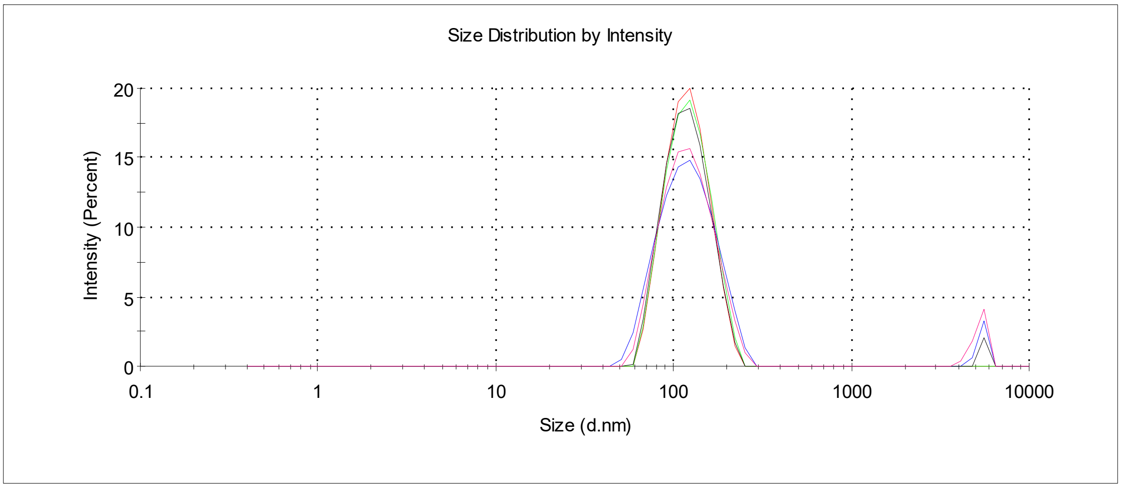

Figure S51. Size distribution of the co-interpolyelectrolyte associates AP[5]A/STC[4]A, where 1:2 molar ratio, the concentration of initial solution AP[5]A/STC[4]A was 3 × 10^{-5} M, final volume was 1 mL). Each line in figure is one measurement from six.

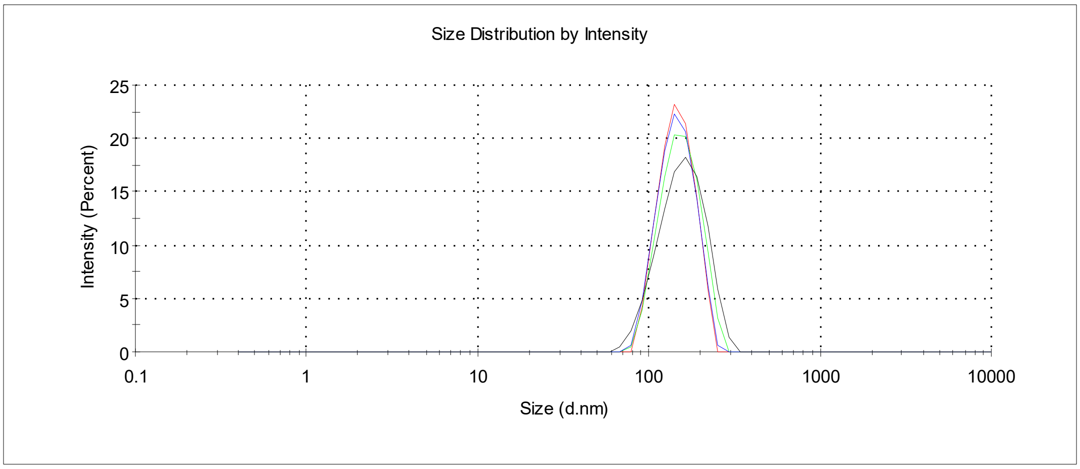

Figure S52. Size distribution of the co-interpolyelectrolyte associates AP[5]A/STC[4]A (1:2 molar ratio, concentration of AP[5]A solution was 3 × 10^{-6} M). Each line in figure is one measurement from four.

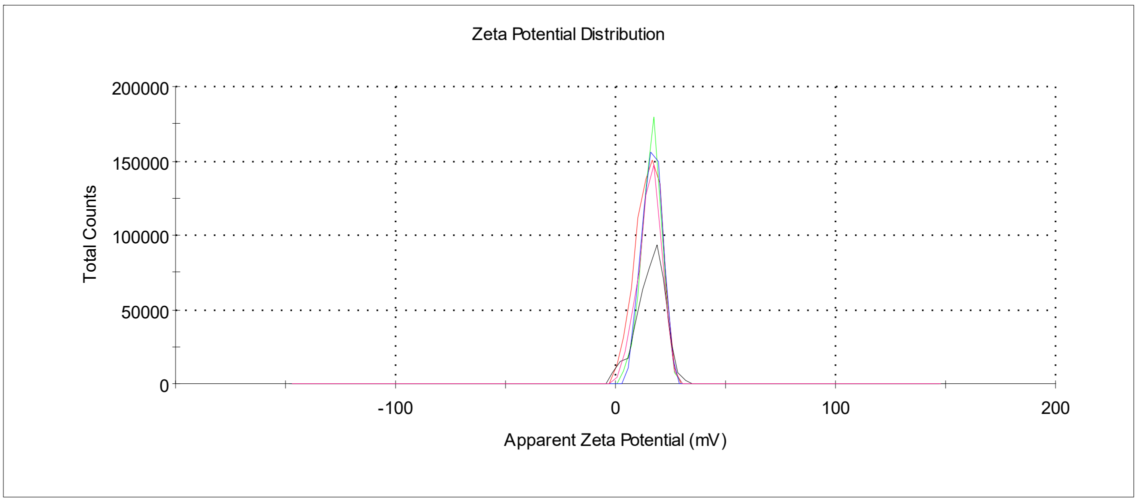

Figure S53. Zeta potential distributions of the associates between co-interpolyelectrolyte associate AP[5]A/STC[4]A (1:2 molar ratio, concentration of AP[5]A solution was 3 × 10^{-5} M). Each line in figure is one measurement from six.

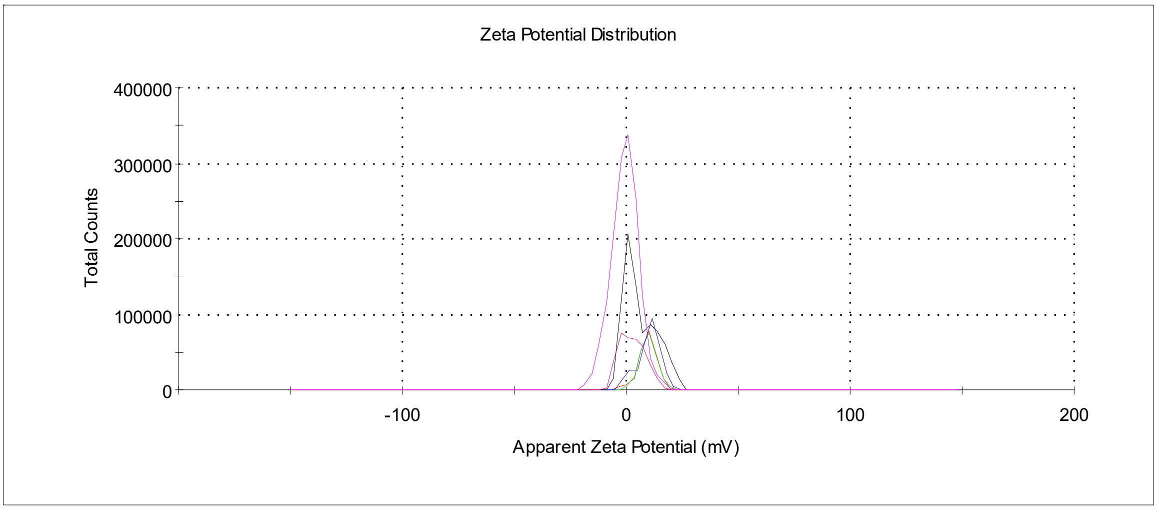

Figure S54. Zeta potential distributions of the associates between co-interpolyelectrolyte associate AP[5]A/STC[4]A (1:2 molar ratio, concentration of AP[5]A solution was 3×10^{-6} M). Each line in figure is one measurement from six.
